# Supplementary material for: Safety and immunogenicity of Ad5-nCoV immunization after three-dose priming with inactivated SARS-CoV-2 vaccine in Chinese adults
Source: Nat Commun. 2023 Aug 8;14:4757. doi: 10.1038/s41467-023-40489-2 (PMC10409730; doi:10.1038/s41467-023-40489-2)
Supplement: Supplementary file 1 — Supplementary Information [file 41467_2023_40489_MOESM1_ESM.pdf]

Supplementary information: Safety and immunogenicity of Ad5-nCoV immunization after three-dose priming with inactivated SARS-CoV-2 vaccine in Chinese adults

Hangjie Zhang[1,#], Nani Xu[2,#], Yu Xu[3], Pan Qin[2], Rongrong Dai[4], Bicheng Xu[3], Shenyu Wang[1], Linling Ding[1], Jian Fu[1], Shupeng Zhang[3], Qianhui Hua[5], Yuting Liao[6], Juan Yang[6], Xiaowei Hu[2,9], Jianmin Jiang[1,4,7,*], Huakun Lv[1,7,*]

[1]Department of Immunization Program, Zhejiang Provincial Center for Disease Control and Prevention, Hangzhou, 310057, China

[2]Xihu District Center for Disease Control and Prevention, Hangzhou, 310007, China

[3]CanSino Biologics, Tianjin, 300457, China

[4]School of Public Health, Hangzhou Medical College Hangzhou, 310053, China

[5]School of Medicine, Ningbo University, Ningbo, 315211, China

[6]School of Public Health, Xiamen University, Xiamen, 361005, China

[7]Key Lab of Vaccine, Prevention and Control of Infectious Disease of Zhejiang Province, Hangzhou, 310057, China

[#]These authors contributed equally: Hangjie Zhang, Nani Xu.

[*]These authors jointly supervised this work: Huakun Lv, Xiaowei Hu, Jianmin Jiang.

e-mail: hklv@cdc.zj.cn.

Supplementary Methods

SARS-CoV-2-specific IgG assay

The commercial anti-SARS-CoV-2 RBD-IgG ELISA detection kit (Vazyme Medical Technology, Nanjing, China) was employed to measure the levels of IgG against SARS-CoV-2 Receptor binding domain (RBD). Briefly, the serum specimen was diluted 3-fold with the sample diluent from 1:10 serially, in addition to the test wells, two negative control wells were included on each plate. After a 30-min incubation at 37°C away from direct light, each well was washed five times with diluted washing buffer, then filled with 100 μL of enzyme-labelled reagents and incubated again under the same conditions. After being washed as described above, each well was filled with 50 μL of chromogen solution A followed by 50 μL of chromogen solution B and then incubated at 37°C for 15 min. Finally, 50 μL of stop solution was added into each well, and the optical density (OD) of each well was measured via dual wavelength detection (at 450 nm/600–650 nm) on a spectrophotometer. The maximum dilution was the titer of the sample. The positive cutoff values for RBD-specific IgG antibodies were defined as titers of 1:90. The titer was converted to relative units per milliliter (RU/ml) with reference to the WHO international standard for anti-SARS-CoV-2 immunoglobulin (NIBSC code 20/136). The positive RBD-specific IgG response was defined as a concentration ≥100 RU/ml.

Pseudovirus-based neutralization test

Serum samples were also quantified for their content of SARS-CoV-2-neutralizing antibodies to Omicron BA.4/5 using the pseudovirus-based virus neutralization test (a vesicular stomatitis virus pseudovirus system that expresses the spike glycoprotein). Briefly, serum samples and a positive or negative reference sample were each diluted 3 times with phosphate-buffered saline combined with 50 μl of pseudovirus diluent per well in a 96-well plate. The mixed sample/pseudovirus was incubated at 37°C and 5% CO2 for 1 h. A 2 × 10^5/ml BHK-21-ACE2 cell suspension was added to each well of the plate containing the sample/pseudovirus mixture, then the plate was incubated in a 37°C and 5% CO2 cell incubator for 48 h. Finally, the number of green-fluorescence-protein-positive cells per well was read with a porous plate imager (Tecan, Shanghai, SparkCyto). 50% neutralization titer (NT50, the reciprocal of the dilution at 50% inhibition) calculated using the Reed-Muench method with a positive cutoff NAb titer ≥1:80. Seroconversion was defined as at least a four-fold increase in antibody levels over the baseline values.

Supplementary Fig.1

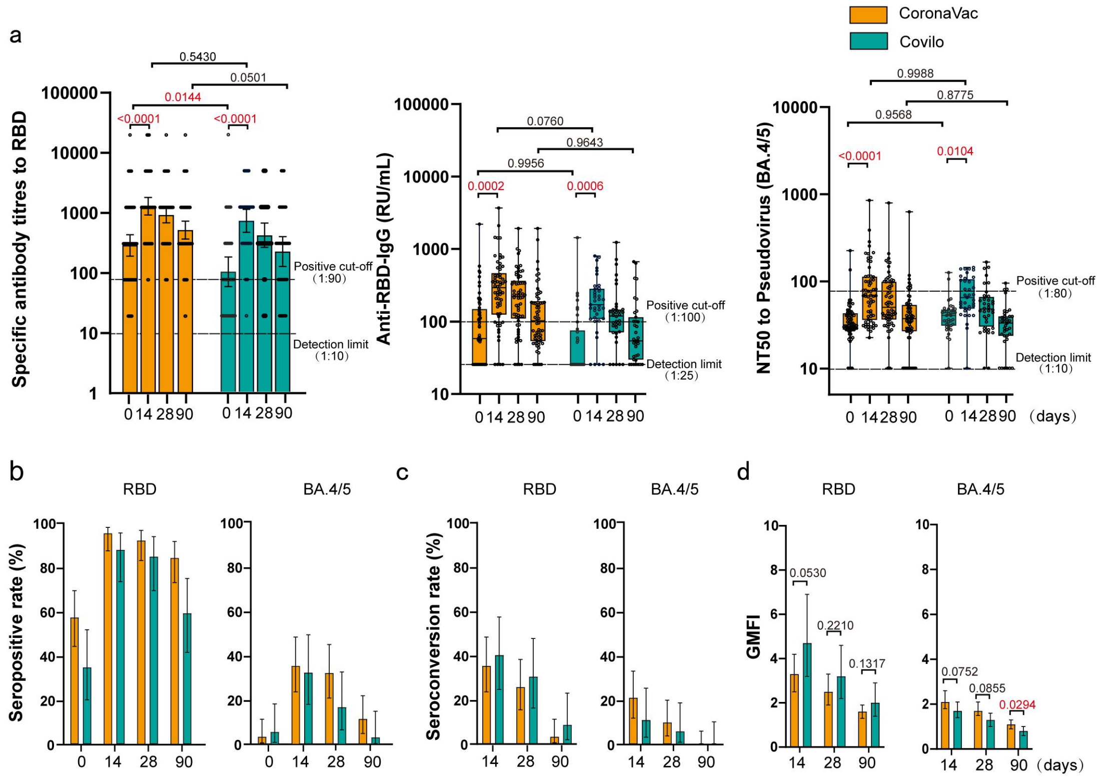

Figure Legend

Supplementary Fig.1 Antibody responses of sub-group of booster vaccination with inactivated vaccine. **(a)** GMT and GMC of SARS-CoV-2 RBD-specific IgG antibodies, and GMT of pseudovirus-neutralizing antibodies to Omicron BA.4/5 on day 0 (before booster vaccination) and days 14, 28, and 90 (after booster vaccination) in sub-group given CoronaVac (N=62, 62, 62, 62) or Covilo (N=37, 37, 36, 35). **(b-d)** Seropositive rates (%) **(b)**, seroconversion rates (%) **(c)**, and GMFI **(d)** of RBD-specific antibodies and pseudovirus-neutralizing antibody 14, 28, and 90 days after booster compared with baseline. Data were shown as Geometric mean ± 95%CI or as box and whiskers in (a), indicating median (middle line), 25th, 75th percentile (box), mean ± 95%CI in (b-d). Seroconversion was defined as at least a 4-fold increase in antibody titers at different time points after booster compared with baseline (day 0). NT50 = 50% neutralization titer. Statistical significance was determined by two-tailed Student's t-test and one-way ANOVA with Tukey's multiple comparisons test. $P < 0.05$ was considered statistically significant and marked with red color. 95% CI: 95% confidence interval, GMT: geometric mean titer, GMC: geometric mean concentration, GMFI: geometric mean fold increase. Source data are provided as a Source Data file.

Supplementary Table 1 Baseline characteristics of enrolled participants of sub-group of inactivated SARS-CoV-2 vaccine booster.

**Variable[a]**	**CoronaVac (n=62)**	**Covilo (n=37)**	**p**
**Sex**			0.09
Male	30(48.4)	24(64.9)	
Female	32(51.6)	13(35.1)	
**Age**			<0.001
18-59 years	46(74.2)	13(35.1)	
≥60 years	16(25.8)	24(64.9)	
Median age (IQR), years	44.0(36.8,60.0)	63.0(42.8,66.5)	
**Body mass index (kg/m2)**			0.426
≤18.4	0(0)	1(2.7)	
18.5-24.9	42(67.8)	22(59.5)	
25.0-29.9	17(27.4)	13(35.1)	
≥30.0	3(4.8)	1(2.7)	
**Time interval since the last priming dose of inactivated vaccine, months**			<0.001
Median (IQR)	6.8(6.5,7.0)	6.4(6.2,6.6)	
**Underlying chronic diseases[b]**			0.294
Yes	19(30.6)	7(18.9)	
No	43(69.4)	30(81.1)	

[a]Data are the number of participants (%), or median (IQR). [b]Underlying chronic diseases included cardiovascular and cerebrovascular diseases, hypertension, and chronic obstructive pulmonary disease. Comparisons were analyzed by Fisher's exact test or Chi-squared test. P-values of less than 0.05 were considered statistically significant.

Supplementary Table 2 RBD-specific IgG antibodies and pseudovirus-neutralizing antibodies to Omicron BA.4/5 in sub-group of inactivated SARS-CoV-2 vaccine booster.

Variable	CoronaVac				Covilo			
	Day 0 (n=62)	Day 14 (n=62)	Day 28 (n=62)	Day 90 (n=62)	Day 0 (n=37)	Day 14 (n=37)	Day 28 (n=36)	Day 90 (n=35)
**Anti-RBD-IgG**								
GMT	85.3 (61.65-118.1)	279.7 (214.2-365.4)	214.4 (138.6-209.8)	135.3 (102.9-177.8)	38.0 (24.3-59.7)	178.2 (125.7-252.6)	114.9 (79.55-165.9)	70.0 (44.68-109.7)
GMC (RU/mL)	136.4 (69.1-203.8)	400.9 (270.6-531.1)	270.6 (200.8-340.3)	180.3 (111.2-249.4)	90.0 (19.4-160.7)	220.2 (159.1-281.4)	172.2 (98.64-245.7)	112.9 (62.09-163.8)
Seropositive rate (%)	58.1 (44.8-70.5)	96.8 (88.8-99.6)	93.5 (84.3-98.2)	85.5 (74.2-93.1)	35.1 (20.2-52.5)	89.2 (74.6-97.0)	86.1 (70.5-95.3)	60.0 (42.1-76.1)
Seroconversion rate (%)	NA	35.5 (23.7-48.7)	25.8 (15.5-38.5)	3.2 (0.4-11.2)	NA	40.5 (24.8-57.9)	30.6 (16.3-48.1)	8.6 (1.8-23.1)
GMFI	NA	3.3 (2.5-4.2)	2.5 (1.9-3.3)	1.6 (1.3-1.9)	NA	4.7 (3.2-6.9)	3.2 (2.2-4.6)	2.0 (1.4-2.9)
**Neutralizing antibodies to Pseudovirus (BA.4/5)**								
GMT	32.2 (28.4-36.6)	68.6 (56.8-82.8)	55.4 (46.2-66.4)	34.0 (28.1-41.3)	37.3 (32.5-42.9)	63.1 (52.7-75.6)	45.9 (37.2-56.5)	28.5 (23.2-35.1)
Seropositive rate (%)	3.2 (0.4-11.2)	35.5 (23.7-48.7)	32.3 (20.9-45.3)	11.3 (4.7-21.9)	5.4 (0.7-18.2)	32.4 (18.0-49.8)	16.7 (6.4-32.8)	2.9 (0.1-14.9)
Seroconversion rate (%)	NA	21.0 (11.7-33.2)	9.7 (3.6-19.9)	0.0 (0.0-5.8)	NA	10.8 (3.0-25.4)	5.6 (0.7-18.7)	0.0 (0-10.0)
GMFI	NA	2.1 (1.8-2.6)	1.7 (1.5-2.1)	1.1 (0.9-1.3)	NA	1.7 (1.4-2.1)	1.3 (1.0-1.6)	0.8 (0.6-1.0)

Supplementary Table 3 Baseline characteristics of enrolled participants of sub-group of booster vaccination with Ad5-nCoV.

Variable[a]	CoronaVac (n=68)	Covilo (n=32)	p
**Sex**			0.863
Male	37(54.4)	18(56.3)	
Female	31(45.6)	14(43.7)	
**Age**			<0.001
18-59 years	51(75.0)	9(28.1)	
≥60 years	17(25.0)	23(71.9)	
Median age (IQR), years	41.5(36.8,57.0)	63.0(53.0,69.8)	
**Body mass index (kg/m2)**			0.226
≤18.4	4(5.9)	0(0)	
18.5-24.9	36(52.9)	18(56.3)	
25.0-29.9	24(35.3)	10(31.2)	
≥30.0	4(5.9)	4(12.5)	
**Time interval since the last priming dose of inactivated vaccine, months**			<0.001
Median (IQR)	6.8(6.6,7.0)	6.4(6.1,6.7)	
**Underlying chronic diseases[b]**			0.029
Yes	17(25.0)	15(46.9)	
No	51(75.0)	17(53.1)	

[a]Data are the number of participants (%), or median (IQR). [b]Underlying chronic diseases included cardiovascular and cerebrovascular diseases, hypertension, and chronic obstructive pulmonary disease. Comparisons were analyzed by Fisher's exact test or Chi-squared test. P-values of less than 0.05 were considered statistically significant.

Supplementary Table 4 RBD-specific IgG antibodies and pseudovirus-neutralizing antibodies to Omicron BA.4/5 in sub-group of booster vaccination with Ad5-nCoV.

Variable	CoronaVac				Covilo			
	Day 0 (n=68)	Day 14 (n=68)	Day 28 (n=68)	Day 90 (n=68)	Day 0 (n=32)	Day 14 (n=32)	Day 28 (n=32)	Day 90 (n=30)
**Anti-RBD-IgG**								
GMT	83.0 (61.6-111.9)	2206.0 (1682.0-2891.0)	1649.0 (1253.0-2170.0)	1175.0 (892.9-1545.0)	38.2 (25.9-56.1)	2348.0 (1580.0-3488.0)	1480.0 (1034.0-2116.0)	752.8 (465.4-1218.0)
GMC (RU/mL)	126.9 (90.1-163.7)	2949.0 (2184.0-3714.0)	2181.0 (1544.0-2819.0)	1390.0 (834.5-1945.0)	59.0 (25.0-93.0)	2870.0 (1760.0-3980.0)	2056.0 (1284.0-2827.0)	1014.0 (558.5-1470.0)
Seropositive rate (%)	62.2 (53.7-77.2)	100.0 (94.7-100.0)	100.0 (94.7-100.0)	100.0 (94.7-100.0)	31.3 (16.1-50.0)	100.0 (89.1-100.0)	100.0 (88.8-100.0)	100.0 (88.4-100.0)
Seroconversion rate (%)	NA	89.7 (79.9-95.8)	86.8 (76.4-93.8)	80.9 (69.5-89.4)	NA	93.8 (79.2-99.2)	93.5 (78.6-99.2)	86.7 (69.3-96.2)
GMFI	NA	26.6 (19.4-36.3)	19.9 (14.4-27.5)	14.2 (10.5-19.0)	NA	61.6 (37.2-101.8)	41.3 (25.7-66.3)	20.1 (12.6-32.2)
**Neutralizing antibodies to Pseudovirus (BA.4/5)**								
GMT	31.4 (28.0-35.1)	234.5 (183.8-299.1)	176.0 (137.8-224.8)	110.3 (87.66-138.9)	27.3 (22.4-33.3)	217.4 (150.4-314.3)	135.4 (90.2-203.3)	79.4 (57.7-109.2)
Seropositive rate (%)	1.5 (0.04-7.9)	80.9 (69.5-89.4)	76.5 (64.6-85.9)	64.7 (52.2-75.9)	0.0 (0.0-10.9)	75.0 (56.6-88.5)	71.0 (52.0-85.8)	53.3 (34.3-71.7)
Seroconversion rate (%)	NA	72.1 (59.9-82.3)	61.8 (49.2-73.3)	38.2 (26.7-50.8)	NA	78.1 (60.0-90.7)	56.1 (33.1-69.8)	40.0 (22.7-59.4)
GMFI	NA	7.5 (5.9-9.4)	5.6 (4.4-7.1)	3.5 (2.8-4.4)	NA	8.0 (5.5-11.7)	5.0 (3.4-7.4)	2.9 (2.0-4.0)

Study on heterologous prime-boost immunization of recombinant COVID-19 vaccine (Ad5-nCoV) based on three doses of inactivated COVID-19 vaccine

Protocol Number: CS—NCOVIM—ZJ001

Sponsor: Zhejiang Provincial Center for Disease Control and Prevention

Version: 1.1, Version Date: April 28th, 2022

Protocol Title	Study on heterologous prime-boost immunization of recombinant COVID-19 vaccine (Ad5-nCoV) based on three doses of inactivated COVID-19 vaccine
Title elaboration	Safety and immunogenicity of heterologous booster immunization with Ad5-nCoV after three-dose priming with inactivated SARS-CoV-2 vaccine in Chinese adults: a randomized, double-blind, parallel-controlled trial
Protocol number	
Investigational vaccine	recombinant COVID-19 vaccine (adenovirus type 5 vector) (hereinafter referred to as Ad5-nCoV)<br>Inactivated COVID-19 vaccine (Vero cell) (hereinafter referred to as ICV)
Protocol date	April 28th, 2022
version number	1.1
Principal Investigator	Hua-Kun Lu
Main editor	Hang-jie Zhang

Copyright 2022. Unauthorized reproduction or use is prohibited.

Statement by Principal Investigator

I agree:

- ✧ Take the responsibility of the main investigator of this clinical research.
- ✧ Ensure that this research is carried out according to the experimental scheme and the revised scheme agreed by all parties.
- ✧ Ensure that the personnel involved in this research master the information of experimental vaccine and the responsibilities and obligations related to the research specified in the experimental plan.
- ✧ Ensure that without the review and written approval of the ethics committee, no changes will be made to the trial protocol, unless it is necessary to eliminate the immediate harm to the subjects or to comply with the requirements of the pharmaceutical administration department (for example, those involving administrative management).
- ✧ I have a complete grasp of the correct method of using the experimental vaccine described in the protocol, including but not limited to the following: the current researcher's manual or equivalent documents.
- ✧ I am familiar with, and will abide by, the Good Practice of Clinical Trials of Drugs and all relevant regulatory requirements.

Protocol Title	Study on heterologous prime-boost immunization of recombinant COVID-19 vaccine (Ad5-nCoV) based on three doses of inactivated COVID-19 vaccine
Title elaboration	Safety and immunogenicity of heterologous booster immunization with Ad5-nCoV after three-dose priming with inactivated SARS-CoV-2 vaccine in Chinese adults: a randomized, double-blind, parallel-controlled trial
Protocol number	CS—NCOVIM—ZJ001
Protocol date	April 28th, 2022
version number	1.1
Principal Investigator	Name: Hua-Kun Lu<br>Title: Chief Physician<br>Position: Director<br>Unit: Zhejiang Provincial Center for Disease Control and Prevention.<br>Address: No.3399, Binsheng Road, Binjiang District, Hangzhou, Zhejiang Provincial, China<br>Postcode: 310051

	Tel: 0571-87115133<br>E-mail：hklv@cdc.zj.cn	
Principal investigator (signature)		Signature date:

Program revision record

No.	Original version number/version date/revision part	Current version number/version date/revision description

Scheme abstract

Protocol Title	Clinical observation and study on heterologous prime-boost immunization of recombinant COVID-19 vaccine (Ad5-nCoV) based on three doses of inactivated COVID-19 vaccine
Title elaboration	On the immunogenicity and safety of heterologous prime-boost immunization of recombinant COVID-19 vaccine (Ad5-nCoV) was carried out in adults aged 18 and above who had completed three doses of inactivated COVID-19 vaccine: a randomized, observer-blind, parallel controlled clinical observation study
prevent disease	Prevention of COVID-19 caused by SARS-CoV-2 i
Research population	Adults over 18 years old
Number of subjects	200 subjects
Research purposes	Main purpose:<br>To evaluate the immunogenicity and safety of a heterologous rime-boost immunization of recombinant COVID-19 vaccine (Ad5-nCoV).<br>Secondary purpose:<br>To evaluate the immunogenicity persistence of a heterologous prime-boost immunization of recombinant COVID-19 vaccine (Ad5-nCoV).
Research site	Hangzhou Xihu District Center for Disease Control and Prevention
fundamental principle	COVID-19 global pandemic has posed a serious threat to the life safety and economy of all countries in the world. Vaccine is one of the most effective ways to end this pandemic. At present, the vaccines against COVID-19 are mainly inactivated vaccine, viral vector vaccine, live attenuated vaccine, recombinant protein vaccine and nucleic acid vaccine.<br>A large number of foreign literatures and studies have proved that vaccines of different technical routes can complement each other through heterologous prime-boost immunization , and heterologous prime-boost immunization strategy is conducive to obtaining a sustained immune response, which can greatly improve and improve the immune effect of vaccines, enhance the body's immunity to mutant Covid-19, reduce the pressure of epidemic prevention and control, and is expected to block the spread of the epidemic. At present, many countries in the world have approved the heterologous prime-boost immunization.<br>In the early stage, we carried out a heterologous study with Ad5-nCoV vaccine as booster, that is to say, the third dose of homologous inactivated vaccine or heterologous Ad5-nCoV vaccine was given at intervals of 3-6 months among people who had been vaccinated with two doses of inactivated vaccine. It was found that heterologous prime-boost immunization can significantly improve the level of neutralizing antibody

	against the real virus after immunization. The Ad5-nCoV vaccine, as the third injection of heterologous prime-boost immunization , induced 5.9 and 6.7 times higher neutralizing antibodies against the original virus variants and Delta variants than that of the third injection of homologous COVID-19 inactivated vaccine, respectively, and induced a high level of Th1-biased cellular immune response. The results showed that intramuscular injection of recombinant Ad5-nCoV vaccine significantly improved the strength of immune response, the breadth and persistence of neutralizing antibody, and formed complementary advantages.<br>On February 19, 2022, with the approval of the joint prevention and control mechanism of the State Council, the National Health and Health Commission has begun to deploy the heterologous prime-boost immunization. People over 18 years old who have been vaccinated with the inactivated vaccines of Sinopharm Beijing Company, Wuhan Company and Beijing Kexing Company for 6 months can use one dose of the adenovirus vector vaccine of Baccino for heterologous prime-boost immunization. However, more than 70% of adults in China have completed the homologous third-dose inactivated vaccine. However, at present, the epidemic situation in China is still developing, especially recently, many provinces and cities in China are experiencing local outbreaks of Omicron variants. According to a clinical trial in the First Affiliated Hospital of Sun Yat-sen University in Guangzhou, 38 first-line medical staff can observe that the serum neutralizing antibody (NAb) against Omicron variants' cross-reaction after the fourth dose of inactivated vaccine is increased, but the degree is low. However, the geometric mean titer (GMNT) of Omicron variants did not change significantly. According to the further research results, the titers of anti-RBD antibodies increased less after four doses of inactivated vaccine, but the titers of anti-NTD antibodies increased more. Because wild-type virus variants can induce NAbs through N-terminal domains (NTD) and receptor visions and (RBD), the cross-neutralizing antibody of Omicron variants mainly targets RBD. Therefore, strengthening four doses of inactivated vaccine has no strong advantage in preventing the epidemic caused by Omicron variants . Therefore, it is urgent to explore the follow-up fourth-dose immunization strategy for people who have been vaccinated with homologous third-dose inactivated vaccine, so as to provide scientific basis for the optimization of the existing COVID-19 vaccine immunization strategy.
Investigational vaccine	Vaccine 1: recombinant COVID-19 vaccine (adenovirus type 5 vector) (hereinafter referred to as Ad5-nCOV).<br>Production unit: Institute of Biological Engineering, Institute of Military Medicine, Academy of Military Sciences/Kangxinuo Bio-stock Company<br>Specification: 0.5ml/ bottle (5×10^{10}VP)

	Dosage: 0.5ml(5×10^{10}VP)<br>Approach: intramuscular injection of deltoid muscle in the lateral upper arm.<br>Vaccine 2: inactivated COVID-19 vaccine (Vero cell) (hereinafter referred to as ICV)<br>Production unit: Beijing Kexing Zhongwei Biotechnology Co., Ltd./Sinopharm Zhongsheng Beijing Biological Products Research Institute Co., Ltd.<br>Specification: 0.5ml/ bottle.<br>Dosage: 0.5ml<br>Approach: intramuscular injection of deltoid muscle in the lateral upper arm.<br>Storage and transportation conditions: It should be stored and transported at 2~8℃ in the dark and protected from freezing.
Trial design	Study design:<br>Randomized, double-blind, and parallel controlled clinical study.<br>Calculation of sample size:<br>(1) Hypothesis 1： The antibody level of Ad5-nCoV group is not inferior to that in the ICV group at Day14 after the boost vaccination.<br>(2)Hypothesis 2： The antibody level of Ad5-nCoV group is superior to that in the ICV group at Day14 after the boost vaccination.<br>6~9 months after vaccination with 3 doses inactivated vaccine ICV, booster immunization was carried out. People aged 60-80 account for about 40%. Subjects were randomly assigned to Ad5-nCoV group or ICV group according to 1:1. Ad5-nCoV group is compared with ICV group. After hypothesis 1 is established, hypothesis 2 can be further statistically inferred. According to the reported research, it is found that the antibody level after immunization is equivalent to the peak antibody level after three doses of inactivated vaccine after six months of immunization. Therefore, it is estimated that the antibody level in ICV group will be 1: 150 (after 10 is the bottom log =2.18) after 14 days of immunization, assuming that the Ad5-nCoV can reach at least 1: 300 after heterologous prime-boost immunization (after 10 is the bottom log = 2.18).<br>Hypothesis 1： The one-sided of the test level α = 0.025, the ratio of ad5-ncov group to ICV group is 1: 1, the non-inferiority limit of GMT ratio is 0.67 (after 10 is the bottom log =-0.174), and the actual GMT ratio of the two groups is 2.00. When the sample size of each group is 80 people, there are 160 people in both groups, the calculated power of the test is 99.86%, which is in line with.<br>Hypothesis 2： The one-sided of the test level α = 0.025, and the ratio of the two groups is 1:1, when the sample size of each group is 80, the total number of the two groups is 160, and the GMT level of the Ad5-nCoV-IM group after immunization is better than that of the ICV group, the estimated power of the test is 88.16%, which meets the test requirements.<br>To sum up, considering that the two hypotheses are met, the shedding rate of the subjects in the experiment (20.00%) and the random block group,

	the sample size of the Ad5-nCoV-IM group and the ICV group are set to be 100 people each, and the two groups are 200 people in total. The sample size of subjects in each group according to age distribution is arranged in Table 1 below.

Table 1 Design and sample size of each group

Test grouping	Age group (years)	group	Sample size (example)
Ad5-nCoV	18~59	A1	60
	60-80	A2	40
ICV	18~59	B1	60
	60-80	B2	40
total			200

Random:

Subjects were stratified randomly according to age (18-59 years old and 60-80 years old). Subjects who have completed 3 doses of inactivated Covid-19 vaccines were randomly vaccinated with Ad5-nCoV or ICV at the ratio of 1:1 in the same age group. An independent randomizing professional generates a random list of subjects through SAS 9.4 or above, and imports it into the interactive response technology (IRT) system, which can only be accessed by authorized personnel. Non-blind personnel of authorized research centers can obtain grouping information of subjects through IRT system, and use research vaccines of corresponding groups according to grouping information.

In this study, non-blind people were set up to distribute, configure and vaccinate. Non-blind people do not participate in other field work except distribution, configuration and vaccination, and other researchers remain blind.

Research plan:

All subjects need to use diary card for systematic safety observation within 14 days after vaccination. All subjects need to complete the blood sample collection on the day of vaccination, 14 days, 28 days, and 3 months after vaccination.

Research endpoint	**Main endpoint indicators:**<br>1) Incidence of Adverse Reactions (AR) in each group from 0 to 14 days after the boost immunization;<br>2) Geometric mean concentration (GMC)/geometric mean titer (GMT), seroconversion/seropositive rates, and geometric mean fold increase (GMFI) of antibodies that bind SARS-CoV-2-specific RBD and pseudovirus-neutralizing antibodies against Omicron BA.4/5 on day 14

<table>
<tr>
<td></td>
<td>post-booster dose.
Secondary endpoint indicators:
Immunogenicity endpoint indicators :
1) Geometric mean concentration (GMC)/geometric mean titer (GMT), seroconversion/seropositive rates, and geometric mean fold increase (GMFI) of antibodies that bind SARS-CoV-2-specific RBD and pseudovirus-neutralizing antibodies against Omicron BA.4/5 on day 28 to 90 days after vaccination.
Safety endpoint indicators :
2) Incidence rate of adverse reactions/events in subjects from 0 to 28 days after the boost immunization;
3) Incidence of serious adverse events (SAE) in subjects within 28 days after the boost immunization.
Exploratory endpoint indicators:
1) Influencing factors (including age, sex, BMI, and chronic diseases) to adverse reactions and immunogenicity.</td>
</tr>
<tr>
<td>Scheduled site visits</td>
<td>This study includes five visits, namely V1 (day 0), V2 (day 14), V3 (day 28), and V4 (month 3).
V1: Informed consent, screening of subjects, random grouping, blood sample collection before immunization, vaccination, observation, distribution of diary card (within 14 days);
V2: Observe adverse events, review the diary card (within 14 days), distribute the diary card (after 14 days), and collect blood samples;
V3: Observe adverse events, review the diary card (after 14 days), and collect blood samples;
V4: Adverse event observation and blood sample collection.
<table>
<tr><td>Visit No.</td><td>V1</td><td>V2</td><td>V3</td><td>V4</td></tr>
<tr><td>Visit interval</td><td>D-7-D0</td><td>D14</td><td>D28</td><td>The 3th month</td></tr>
<tr><td>Time window</td><td>0 days</td><td>+3 days</td><td>+5 days</td><td>+30 days</td></tr>
<tr><td>informed consent</td><td>●</td><td></td><td></td><td></td></tr>
<tr><td>Registration/verification of identity information</td><td>●</td><td>●</td><td>●</td><td>●</td></tr>
<tr><td>Physical examination (blood pressure, height, weight, temperature)</td><td>●</td><td></td><td></td><td></td></tr>
<tr><td>Urinary pregnancy (women who have not yet reached menopause)</td><td>●</td><td></td><td></td><td></td></tr>
<tr><td>Medical history inquiry and admission screening</td><td>●</td><td></td><td></td><td></td></tr>
<tr><td>Random number assignment</td><td>●</td><td></td><td></td><td></td></tr>
<tr><td>blood specimen collection</td><td>10ml</td><td>10ml</td><td>10ml</td><td>10ml</td></tr>
</table>
</td>
</tr>
</table>

<table>
<tr>
<td></td>
<td>
<table>
<tr><td>Vaccination[a]</td><td>●</td><td></td><td></td><td></td></tr>
<tr><td>Distribution diary card (within 14 days)</td><td>●</td><td></td><td></td><td></td></tr>
<tr><td>Distribution of diary card (after 14 days)</td><td></td><td>●</td><td></td><td></td></tr>
<tr><td>Audit diary card</td><td></td><td>●</td><td>●</td><td></td></tr>
<tr><td>Safety observation[b]</td><td>●</td><td>●</td><td>●</td><td></td></tr>
<tr><td>SAE observation and report[c]</td><td>●</td><td>●</td><td>●</td><td>●</td></tr>
</table>

a) If the researcher thinks that the health condition of the subjects on the day of vaccination is temporarily unsuitable for vaccination, the vaccination can be delayed within one week;
b) Observe and record all adverse events from day 0 to day 28 after vaccination, whether related to vaccine or not;
c) The researcher shall fill in the "serious adverse events Report Form" to complete the first report within 24 hours after learning that the subject has SAE, and complete the summary report after the event.
d) If the screening period is divided over two days, blood pressure and temperature must be re-measured on the day of enrollment.
</td>
</tr>
<tr>
<td>Research suspension criteria and early termination criteria</td>
<td>In case of any of the following circumstances, the trial will be suspended, and the researcher shall hold an expert group meeting to decide whether to terminate the clinical trial in advance:
− There was one case of grade 4 or above adverse reaction possibly related to vaccination;
− One case of serious or life-threatening SUSAR occurred;
− The number of subjects with adverse reactions with severity of Grade 3 and symptoms lasting for > 48 hours and not relieved to Grade 1 or 2 exceeded 15% of the vaccinated subjects;
One of the following situations occurs, the study is terminated:
− The ethics committee requires the complete termination of the experiment and explains the reasons;
− The administrative department requires a complete termination of the test and explains the reasons.</td>
</tr>
<tr>
<td>statistical analysis</td>
<td>The first stage analysis:
After 14 days, the results of safety and immunogenicity data were verified, and the first stage analysis was carried out.
Final analysis:
The final analysis was carried out after the results of safety data and immunogenicity data for 3 months were verified.</td>
</tr>
<tr>
<td>Inclusion and exclusion criteria</td>
<td>Inclusion criteria:
1) When screening volunteers aged 18 and above;
2) Get the informed consent of volunteers and sign the informed consent form;</td>
</tr>
</table>

	3) Volunteers are able and willing to comply with the requirements of clinical trial protocol and can complete 3-month follow-up study;<br>4) Three doses of inactivated Covid-19 vaccines have been vaccinated, and the interval between this vaccination and this vaccination is between 6 and 9 months.<br>Exclusion criteria:<br>1) Those who have a medical history or family history of convulsion, epilepsy, encephalopathy and psychosis;<br>2) Those who are allergic to any component of the research vaccine, or those who have severe vaccine allergic reaction, allergic history or asthma history in the past;<br>3) Serious adverse events related to vaccination occurred after vaccination with COVID-19 vaccine in the past;<br>4) Women with positive urine pregnancy test or breast-feeding;<br>5) People with acute febrile diseases, infectious diseases and a history of SARS;<br>6) Underarm temperature > 37.0°C;<br>7) Suffering from serious cardiovascular diseases, such as arrhythmia, conduction block, myocardial infarction, severe hypertension and uncontrollable drugs (systolic blood pressure ≥180mmHg and/or diastolic blood pressure ≥110mmHg when measured on site), etc.;<br>8) Suffering from severe chronic diseases, such as diabetes, thyroid diseases, etc., which are in the progressive stage and cannot be controlled smoothly;<br>9) Congenital acquired angioedema/neuroedema;<br>10) One year before receiving the experimental vaccine, he had urticaria;<br>11) Have asplenia or functional asplenia;<br>12) Suffering from pulmonary dysfunction such as chronic obstructive pulmonary disease and pulmonary fibrosis;<br>13) Have a history of Covid-19 infection/illness;<br>14) In the past 21 days, he has been to medium and high-risk areas or has a history of leaving the country, and has a history of epidemiological contact in COVID-19;<br>15) The researcher's judgment based on various medical, psychological, social or other conditions may be contrary to the experimental plan or affect the informed consent of the subjects.
duration	About 8 months.
Principal Investigator (PI)	Name: Hua-Kun Lu<br>Title: Chief Physician<br>Position: Director<br>Unit: Zhejiang Provincial Center for Disease Control and Prevention.<br>Address: No.3399, Binsheng Road, Binjiang District, Hangzhou, Zhejiang

	Provincial, China<br>Postcode: 310051<br>Tel: 0571-87115133<br>E-mail： hklv@cdc.zj.cn
Jiangsu Provincial Center for Disease Control and Prevention (Jiangsu Public Health Research Institute)	Person in charge: Guo Xiling<br>Unit: Jiangsu Provincial Center for Disease Control and Prevention (Jiangsu Public Health Research Institute)<br>Address: No.172 Jiangsu Road, Gulou District, Nanjing<br>Postcode: 210009<br>Tel: 18915999489<br>E-mail： 1250535183@qq.com
Shanghai aimeishi medicine technology co., ltd	Person in charge: Wang Xuewen<br>SETTING: Shanghai Aimeishi Pharmaceutical Technology Co., Ltd.<br>Unit: Room 1012, No.7, Lane 200, Yunxin Road, Jing 'an District, Shanghai<br>Postcode: 200040<br>Tel: 13761517181<br>Fax: 021-5677-9877<br>E-mail： xuewen.wang@imstat.cn

English abbreviation list

abbreviation	English name	Chinese name
AE	Adverse Event	Adverse event
AR	Adverse Reaction	adverse effect
Ad5	Replication Defective Human Adenovirus Serotype 5	Replication deficient human adenovirus type 5
COVID-19	Corona Virus Disease 2019	COVID-19
eCRF	Electronic Case Report Form	Electronic case report form
ELISA	Enzyme-linked Immunosorbent Assay	Enzyme-linked immunosorbent assay
FAS	Full Analysis Set	Total analysis set
GCP	Good Clinical Practice	Good Clinical Practice
GMC	Geometric Mean Concentration	Geometric average concentration
GMI	Geometric Mean Fold Increase	Geometric average growth multiple
GMP	Good Manufacturing Practice	Good Manufacturing Practice of Medical Products
GMT	Geometric Mean Titre	Geometric average titer
IEC	Independent Ethics Committee	Ethics Committee
ITT	Intent-to-treat	Intentional treatment
NIFDC	National Institute for Food and Drug Control	China Food and Drug Verification Research Institute
NMPA	National Medical Products Administration	the State Drug Administration (SDA)
PPS	Per Protocol Set	Conforming scheme data set
SAE	Serious Adverse Event	serious adverse events
SS	Safety Set	Security analysis set
VP	Virus Particle	Viral particle

TABLE OF CONTENTS

1. Purpose and Introduction 1
2. Research management 2
2.1 responsible unit 2
2.2 Specimen testing unit—Nanjing Novozen Biotechnology Co., Ltd. and Jiangsu Center for Disease Control and Prevention (Jiangsu Public Health Research Institute) 3
2.3 Data management and statistical analysis unit-Shanghai Aimeishi Pharmaceutical Technology Co., Ltd. 3
3. Research background 4
3.1 Disease background 4
3.2 Virus background 4
the most important surface protein of coronavirus, which is related to the transmission ability of the virus. S protein contains two subunits: S1 and S2. S1 mainly contains receptor binding region, which is responsible for the recognition of cellular receptors. S2 contains the basic elements for the membrane fusion process. In the previous development of SARS and MERS vaccines, S protein was used as the most important candidate antigen.3.3 Background of vaccines 5
3.3.1 Pre-clinical immunogenicity evaluation (mouse model: intramuscular injection+mucosal immunization) 7
3.3.2 Pre-clinical protective test 12
3.3.2.1 Ferret model (intramuscular injection+mucosal immunization) 12
3.3.2.2 Rhesus monkey conservation experiment (nebulized inhalation) 13
3.3.3 Pre-clinical safety evaluation 14
3.3.3.1 Single-dose toxicity studies in SD mouses (mucosal immunity) 14
3.3.3.2 Toxicity studies of repeated administration of rhesus monkeys (mucosal immunity, nebulized inhalation) 15
3.3.3.3 Study on repeated administration toxicity of cynomolgus monkeys (intramuscular injection) 16
3.4 Preliminary clinical research results 16
3.4.1 Sequential clinical study of muscle 16
4. Introduction of vaccines 17
5. Test objective 17
5.1 Main purpose 17
5.2 Secondary purpose 17
6. Experiment design 17
6.1 Study endpoint index 18
6.1.1 Main endpoint indicators 18
6.1.2 Secondary endpoint indicators 18
6.1.3 Exploratory endpoint indicators 19
6.2 Sample size estimation 19
6.3 Numbering Rules 20
6.3.1 Screening number 20
6.3.2 Random numbering rules 20

6.4 Blind vaccine method 20
6.4.1 Packaging of vaccine for test 20
6.4.2 Editing blindness 20
6.4.3 Blind bottom preservation 21
6.4.4 Blindness Maintenance 21
6.4.5 Blindness Removal 21
6.4.6 Emergency blind uncovering 21
6.5 Test period 21
6.6 Suspension and early termination of research 21
7. Subjects 22
7.1 Selection of subjects 22
7.2 Inclusion criteria 22
7.3 Exclusion criteria 22
7.4 Criteria for Subjects to Terminate the Test Early 23
7.5 Rejection criteria 23
8. Methods and procedures 23
8.1 Selection of subjects 23
8.2 Informed consent 23
8.3 On-site registration 24
8.4 Physical examination and screening 24
8.5 Subjects were randomly divided into groups. 24
8.6 Vaccine and use 24
8.6.1 Research vaccine 24
8.6.2 Immunization routes and immunization procedures 25
8.6.3 Storage and transportation of vaccines for research 25
8.6.4 Combined medication 25
8.6.5 Organization and management of research vaccines 25
8.7 Follow-up and evaluation of safety 26
8.7.1 Safety observation 26
8.7.2 Contents and indicators of safety observation 26
8.7.3 Outcome of AEs 32
8.7.4 Relationship between AEs and vaccination 32
8.7.5 Treatment of Adverse Reactions/Events 33
8.7.6 serious adverse events's reporting procedure 33
8.7.7 Treatment of pregnancy events 34
8.8 Evaluation of immunogenicity 34
8.8.1 Specimen collection 34
8.8.2 ELISA detection of S-RBD IgG antibody against SARS-CoV-2 35
8.8.3 VOC/VOI cross neutralization detection 35
8.8.4 Serum number 35
8.8.5 Preservation and transportation of specimens 35
8.9 Site Work Flow 35
8.9.1 Overview of visiting process 35
8.9.2 Field personnel grouping and task division 36

8.9.3 Site management......37
8.10 Data management......38
8.10.1 Design and Establishment of Database......38
8.10.2 Data entry......38
8.10.3 Audit of data records......38
8.10.4 Data verification......39
8.10.5 Medical Coding......39
8.10.6 Database Locking......39
8.10.7 External data management......39
8.10.8 eCRF archiving......39
8.11 Statistical analysis......39
8.11.1 Statistical Analysis Plan......39
8.11.2 Research hypothesis......40
8.11.3 Selection of analysis datasets......40
8.11.4 Data statistics method......41
8.11.4.1 Demographic data and baseline characteristics......41
8.11.4.2 Safety analysis......41
8.11.4.3 Immunogenicity analysis......42
8.11.5 Analysis arrangement......43
8.11.6 Analysis software......43
9 Monitoring of clinical trials......43
9.1 Responsibilities of each party......43
9.1.1 Researchers......43
9.1.2 Inspector......43
9.2 Personnel training......44
9.2.1 Field operators......44
9.2.2 Laboratory operators......44
9.2.3 Clinical medical staff......44
9.2.4 Auditor (CRA)......45
9.3 Experimental reagents and methods (specimen testing unit)......45
9.3.1 Reagent Management......45
9.3.2 Experimental Methods......45
9.4 vaccine management......45
9.5 Site and laboratory supervision......46
9.6 Quality control of documents and materials......46
9.6.1 Original data......46
On-the-spot documents of 9.6.1.1 researchers......46
9.6.1.2 Laboratory Documents......46
9.6.2 Case Report Form (eCRF)......47
9.6.3 Preservation of data......47
9.7 Quality Control of Biological Specimens......47
9.7.1 Quality control of biological specimen collection and processing......47
9.7.2 Quality control of biological specimen transportation process......48
9.7.3 Quality control of biological specimen preservation......48

10 risk management plan 48
0.1 safety specification 48
0.2 pharmacovigilance program 48
0.3 risk minimization measures 49
1 timetable 49
2 ethical approval 49
12.1 review and approval 49
12.2 Supervise the following implementation processes 49
12.2.1 Informed consent 49
12.2.2 Confidentiality 49
12.2.3 Potential hazards and minimization of hazards 50
13. Publication and publication of data 50
14. References 50

1. Purpose and Introduction

2019 Novel Coronavirus (SARS-CoV-2) is an unsegmented single-stranded RNA virus, belonging to Canoviridae, Canovirinae. There are six types of coronaviruses known to infect people, including 229E and NL63 of α, OC43 and HKU1 of β, Middle East respiratory syndrome-associated coronavirus (MERS-CoV) and severe acute respiratory syndrome-associated coronavirus (SARS-CoV). 2019 novel coronavirus is the seventh coronavirus that can infect people. Following the outbreak of severe acute respiratory syndrome coronavirus (SARS-CoV) in 2002 and the outbreak of Middle East respiratory syndrome coronavirus (MERS-CoV) in 2012, 2019 novel coronavirus is the third highly pathogenic coronavirus in the past 20 years.

The recombinant COVID-19 vaccine (adenovirus type 5 vector) for inhalation, jointly developed by the Institute of Bioengineering, Institute of Military Medicine, Academy of Military Sciences, and Concino Biotech Co., Ltd., prevents COVID-19 caused by the 2019 novel coronavirus' infection. The vaccine takes replication-defective human adenovirus type 5 as a carrier, can express the specific S protein of 2019 novel coronavirus, and is made by amplification and purification. Pre-clinical studies suggest that both humoral immunity and cellular immunity play an important role in protective immunity. The main function of humoral immunity is to prevent viruses from infecting host cells, while the main function of cellular immunity is to clear virus-infected cells.

On March 16th, 2020, the first phase clinical trial of recombinant novel coronavirus vaccine (adenovirus type 5 vector) was started in Wuhan. The results showed that the low and middle dose vaccines showed good safety in human body, and the high dose vaccines showed clinically tolerable safety. On April 12th, 2020, the second phase clinical trial of recombinant novel coronavirus vaccine (adenovirus type 5 vector) was started in Wuhan. The results showed that low-dose and middle-dose vaccines showed good safety in human body, and the safety of low-dose vaccine was better than that of middle-dose vaccine. Humoral immunity showed that the positive conversion rate of S-RBD protein in low dose group and middle dose group was higher. The positive rate of specific IFN-γ detected by ELISpot in low dose group and middle dose group after vaccinate was significantly higher than that in placebo group[1]. On September 21, 2020, PhaseIIb clinical trial of recombinant COVID-19 vaccine (adenovirus type 5 vector) was started in Taizhou, Jiangsu Province. The immunogenicity and safety of two doses of recombinant COVID-19 vaccine (adenovirus type 5 vector) were evaluated among people aged 6 and above and those who had been vaccinated with recombinant Ebola virus disease vaccine (adenovirus type 5). According to the results of mid-term analysis, 0.3ml for teenagers was safe, and the immunogenicity was similar to 0.5ml for adults aged 18~59.

Phase III clinical trial of recombinant COVID-19 vaccine (adenovirus type 5 vector) was conducted in five countries include Mexico and so on, about 44,000 subjects were vaccinated. The results of mid-term analysis showed that after 14 days of single-dose vaccination, the overall protection effect was 68.8%, the protection rate of the elderly was 67.8%, and the critical protection rate was 95.5%. There was no vaccine-related SAE.

Therefore, the recombinant novel coronavirus vaccine (adenovirus type 5 vector) is safe and effective. The vaccine was approved for conditional marketing by National Medical Products

Administration on February 25th, 2021, and also approved for emergency use by Mexico, Pakistan, Hungary, Chile and other countries in the same period.

According to the results of a clinical study in the First Affiliated Hospital of Sun Yat-sen University, Guangzhou, in which people who are immunized with the 3 doses of inactivated vaccine are given a homologous 4-dose of inactivated vaccine, the level of serum neutralizing antibody against SARS-CoV-2 wild strain dropped sharply by 85% at 26 weeks (about 6 months) after the 3-dose inactivated vaccine against COVID-19, and the level of cross-neutralizing antibody against Omicron variant strain dropped to a lower level. Therefore, the immune persistence of the 3-dose of COVID-19 inactivated vaccine was not ideal. In addition, the study also found that the antibody level after the 4-dose of inactivated vaccine was equivalent to that after the 3-dose of inactivated vaccine, but there was no significant increase[2], so there is an urgent need for new exploration of the fourth dose of immunization strategy. Considering the difference of immune response types and characteristics between inactivated vaccine and recombinant novel coronavirus vaccine (adenovirus type 5 vector), sequential vaccination of these two vaccines may complement each other, improve the speed and quality of immune response, and optimize the existing immunization strategy of COVID-19 vaccine. In order to explore the safety and immunogenicity of one dose of adenovirus type 5 vector vaccine or COVID-19 inactivated vaccine for healthy people over 18 years old who have completed three doses of basic immunization with COVID-19 inactivated vaccine at intervals of 6~9 months, this study is planned. This clinical trial plan is formulated according to the requirements of the Vaccine Administration Law, Drug Registration Administration Measures, Good Practice for Clinical Trial of Drugs (GCP), Technical Guidelines for Clinical Trial of Vaccines, Technical Guidelines for Clinical Comparability Research of Preventive Vaccines and Guidelines for Quality Management of Clinical Trial of Vaccines (for Trial Implementation).

2. Research management

2.1 responsible unit

(1) Participate in the formulation of clinical trial plan and organize the implementation of clinical trial plan;

(2) Assist in the preparation and review of informed consent, vaccination and visit records, diary cards and other field application forms;
(3) Submit ethical audit materials to Independent Ethic Committee, IEC) and obtain approval certificate;
(4) Establish the organization and management system and quality management system of vaccine clinical trials, and carry out training;
(5) Recommend clinical trial sites, and organize and assist site standardization construction;
(6) It has the management mechanism and measures to prevent and deal with emergencies in clinical trials of vaccines, and has the team of SAE emergency treatment experts and the technical ability to deal with SAE;
(7) Organize on-site recruitment and grouping of subjects, organize on-site vaccination and supervise the implementation of on-site work;
(8) Organize and ensure the safe storage and use of experimental vaccines, and organize and manage biological samples;
(9) Organize the follow-up of subjects and the collection of adverse events at the test site, and organize the reporting, investigation and treatment of adverse events;
(10) Complete all forms filling and Electronic Case Report Form, eCRF) entry at the test site;
(11) Confirm the archiving of test data, manage and save test-related data according to GCP requirements until 5 years after the test drug is approved for marketing;
(12) Issue a summary report of clinical trials.

2.2 Specimen testing unit—Nanjing Novozen Biotechnology Co., Ltd. and Jiangsu Center for Disease Control and Prevention (Jiangsu Public Health Research Institute)

(1) Complete the test of clinical research samples according to the prescribed method and issue a test report;
(2) Providing a result judgment reference value;
(3) Issue certification, accreditation, quality control and other laboratory-related qualification certificates.

2.3 Data management and statistical analysis unit-Shanghai Aimeishi Pharmaceutical Technology Co., Ltd.

(1) Participate in the formulation of clinical trial plan;
(2) Write statistical analysis plan according to clinical trial plan;
(3) Randomly coding the experimental vaccine;
(4) Responsible for the establishment and data management of project eCRF;
(5) Make statistics and issue statistical analysis report.

3. Research background

3.1 Disease background

The COVID-19's main symptoms are fever, dry cough and fatigue. A few patients have symptoms such as nasal congestion, runny nose, sore throat, myalgia and diarrhea. Severe patients usually develop dyspnea and/or hypoxemia one week after onset, and in severe cases, rapid progression to acute respiratory distress syndrome, septic shock, refractory metabolic acidosis, haemorrhagic dysfunction and multiple organ failure, etc. It is worth noting that the course of the disease in the severe and critical patients may be moderate to low fever, or even no obvious fever. Some children and newborns showed atypical symptoms, such as diarrhea, vomiting and other digestive tract symptoms, or only mental weakness and shortness of breath. Mild patients only showed low fever, slight fatigue, and no pneumonia. Judging from the current cases, most patients have a good prognosis, while a few patients are in critical condition. The prognosis of the elderly and those with chronic underlying diseases is poor. The clinical process of pregnant women with SARS-CoV-2 is similar to that of patients of the same age.

At present, the main source of infection of the virus is SARS-CoV-2 infected patients, and asymptomatic infected persons may also become the source of infection. People in SARS-CoV-2 are generally susceptible, mainly through respiratory droplets and close contact, but exposure to high concentrations of aerosols in a relatively closed
environment for a long period of time has the potential for aerosol transmission. As SARS-CoV-2 can be isolated from feces and urine, attention should also be paid to aerosol or contact transmission caused by feces and urine to environmental pollution.

3.2 Virus background

Novel coronavirus's disease is an infectious disease caused by novel coronavirus infection. At the end of 2019, novel coronavirus was first found in the case of viral pneumonia in Wuhan, China. On February 11th, 2020, the World Health Organization named the disease COVID-19.

2019 Novel Coronavirus 2019(SARS-CoV-2) belongs to the genus β of coronavirus, with enveloped granules that are round or elliptic, often pleomorphic, with diameters ranging from 60 nm to 140nm. Its genetic characteristics were significantly different from those of SARS-CoV and MERS-CoV. Scientists in China found that the gene sequences of two bats (bat-SL-CoVZC45 and bat-SL-CoVZXC21) in Zhoushan, China have 88% homology[3]. Novel coronavirus, discovered in Wuhan this time, is the seventh coronavirus that can infect humans. It has not been found in humans before.

SARS-CoV-2Coronaviruses belong to the genus Coronavirus in the family Coronaviridae. Coronaviruses are single-stranded RNA viruses with an envelope. They are a large group of viruses that exist widely in nature. Globally, 10% to 30% of upper respiratory tract infections are caused by HCoV-229E, HCoV-OC43, HCoV-NL63 and HCoV-HKU1, which are the second leading cause of the common cold, after rhinoviruses. Middle East Respiratory Syndrome (MERS) and Severe Acute Respiratory Syndrome (SARS), caused by coronavirus, are known to be serious infectious diseases.

The coronavirus genome encodes spike protein (S), envelope protein (E), membrane protein

(M) and nucleoprotein (N) in sequence. Among them, S protein is the most important surface protein of coronavirus, which is related to the transmission ability of the virus. S protein contains two subunits: S1 and S2. S1 mainly contains receptor binding region, which is responsible for the recognition of cellular receptors. S2 contains the basic elements for the membrane fusion process. In the previous development of SARS and MERS vaccines, S protein was used as the most important candidate antigen.

3.3 Background of vaccines

The recombinant novel coronavirus vaccine (adenovirus type 5 vector) is a recombinant novel coronavirus vaccine (adenovirus vector) jointly developed by the Institute of Biological Engineering, Institute of Military Medicine, Academy of Military Sciences, and Kangsino Biological Co., Ltd. Based on the mature recombinant replication-defective human adenovirus type 5 vector platform, it can efficiently express novel coronavirus's target antigen (S protein) in transfected/infected cells, and it is expected that it can induce humoral immunity, cellular immunity and mucosal immunity against novel coronavirus envelope protein after vaccination, thus providing protection for vaccinators.

At present, there are mainly the following kinds of COVID-19 vaccines under research:

Inactivated vaccine: It is composed of complete virus, and its pathogenicity loss still keeps all or part of the immunogenicity of the virus. After vaccinate, virus antigen can stimulate the body to produce immune response, thus achieving protection. Inactivated vaccine needs to go through the following steps: firstly, strain is cultured and screened on suitable cells, and a stable virus with high titer is obtained, which can be used as a seed bank for mass production of vaccines in the future. Candidate vaccines are prepared through the processes of culture, inactivation, purification, etc. The process is relatively simple, which is a traditional and classic vaccine preparation method. The main obstacles lie in two aspects: the research on the pathogenic mechanism and immunological mechanism of Covid-19 is not well studied, and the inactivated whole virus may carry harmful components; Second, at present, the live virus culture is required to be carried out under P3 biosafety conditions, and the production capacity will be limited.

Recombinant subunit vaccine: It is made from effective antigen of virus that can stimulate the organism to produce protective immunity. It is safe and secure, but it is generally small in size and poor in immunogenicity. It needs some new technical means and adjuvants to increase its immunogenicity. And the design and effectiveness evaluation are the key points, and the development cycle is long.

Adenovirus vector vaccine: Replication-deficient human adenovirus type 5 vector vaccine containing 2019-nCoV antigen gene can efficiently express the target antigen of 2019-nCoV in transfected/infected cells, so that the body can produce corresponding humoral immunity and cellular immunity, and can provide effective protection against diseases caused by 2019-nCoV. This vaccine uses the same adenovirus vector platform as the approved recombinant Ebola virus vaccine, and has a certain research and development foundation.

Attenuated influenza virus vector vaccine: the vaccine is vaccinated by nasal instillation, and if successfully developed, it will have a certain effect on improving the vaccination rate. At

present, there are no reports of similar vaccines in other countries around the world.

mRNA: The mRNA of a variety of different antigen sequences targeting the key targets of 2019-nCoV virus is synthesized in vitro, and then delivered to the body and translated into antigen proteins by cells in the body, thus activating the body's immune system and causing specific immune response. The drug mRNA is simple to produce, easy to reform, quick to synthesize and low in cost, but it has the defects of poor stability and too strong immunogenicity.

As of February 6, 2022, 61.4% of the global population has received at least one dose of COVID-19 vaccine. 10.2 billion doses have been administered worldwide, with an average daily dose of 18.22 million. Chile, Canada, Germany, Italy, the United Kingdom, the United States and other middle-and high-income countries have more than 70% of the population fully vaccinated. About 10% of low-income people have received at least one dose of COVID-19 vaccine. Vaccination in COVID-19 of China was officially launched on December 15th, 2020. The initial target population was people aged 18~59 who were engaged in high-risk occupations, and then it was expanded to all people aged 18 and above. According to the figures released by Our World Data, as of February 4, 2022, nearly 1.27 billion people (about 91% of the population) have received at least one dose of COVID-19 vaccine, and the progress ranks first in the world.

According to the statistics of Epidemic Prevention Innovation Alliance (CEPI), as of June, 2021, a number of vaccine sequential vaccination studies (including basic sequential immunization and booster sequential immunization) have been underway in the world, including 13 basic sequential immunization studies and 16 booster sequential immunization studies. At present, some data obtained support that different kinds of vaccines can be mixed, and compared with the same type of vaccines, they can stimulate a higher level of humoral immune response and produce a strong T-cell immune response, and they are highly effective against variants[4-6]. On December 16th, 2021, the World Health Organization (WHO) issued the interim guidelines for vaccine heterologous booster in COVID-19. According to the guideline, based on the existing clinical results and application evidence, compared with the homologous vaccination program using only inactivated vaccine, heterologous vaccination with vector vaccine or mRNA vaccine showed enhanced immunogenicity, and the level of neutralizing antibody was better than that of homologous immunization with inactivated vaccine.

In order to better cope with the epidemic prevention and control, many countries are carrying out booster vaccination. According to the information published by the media, up to now, many countries and regions have adopted or recommended "heterologous sequential immunization" as a booster strategy, such as the United Kingdom, the United States, Canada, Chile, Argentina, Thailand, etc. Among them, the United Kingdom recommends that adenovirus vector vaccine and mRNA vaccine be used alternately as booster immunization, that is, if mRNA vaccine is used for basic immunization, adenovirus vector vaccine is used for booster immunization, and vice versa; In Chile and Chile, inactivated vaccine is used as basic immunization and adenovirus vector vaccine is used as booster immunization. Brazil and UAE all adopt inactivated vaccine as basic immunization, and adenovirus vector vaccine or mRNA vaccine as booster immunization; The United States Food and Drug Administration (FDA) has approved a mixed vaccination program using different kinds of vaccines as boosters. Canada and major European countries have approved the mixed vaccination of adenovirus vector vaccine and mRNA vaccine.

In the early stage, we carried out a clinical trial to immunize people who had been vaccinated with COVID-19 inactivated vaccine with recombinant Ad5 vector COVID-19 vaccine as booster. The third dose of homologous or heterologous booster sequential immunization program was designed from 3 to 6 months after two doses of inactivated COVID-19 vaccine, and the second dose of homologous or heterologous basic sequential immunization program from 1 to 2 months after one dose of inactivated COVID-19 vaccine. It was found that heterologous sequential immunization can significantly improve the neutralizing antibody level of the real virus after immunization: the third-dose heterologous booster immunization program > the second-dose heterologous basic immunization program > three doses inactivated vaccines homologous booster immunization program > two doses inactivated vaccines homologous basic immunization program. The recombinant Ad5 vector COVID-19 vaccine, as the third injection of allogeneic booster immunization, induced 5.9 and 6.7 times higher neutralizing antibodies against the original virus strain and Delta strain than that of the third injection of homologous inactivated vaccine, respectively, and induced a high level of Th1-biased cellular immune response. The results showed that intramuscular injection of recombinant Ad5 vector vaccine significantly improved the strength of immune response, the breadth and persistence of neutralizing antibody, and formed complementary advantages.

On February 19, 2022, with the approval of the joint prevention and control mechanism of the State Council, the National Health and Health Commission has begun to deploy the sequential booster immunization. People over 18 years old who have been vaccinated with the inactivated vaccines of Sinopharm Beijing Company, Wuhan Company and Beijing Kexing Company for 6 months can use one dose of the adenovirus vector vaccine of Baccino for sequential booster immunization. However, more than 70% of adults in China have completed the homologous third-dose inactivated vaccine. However, the peak level of antibody against Wuhan strain in the third-dose inactivated vaccine booster immunization is only 20% of WHO standard serum reference. A recent study found that the level of neutralizing antibody against Wuhan strain 6 months after three doses of inactivated vaccine decreased by about 85% compared with the peak level after the third-dose. Therefore, it is urgent to explore the follow-up fourth-dose immunization strategy for people who have been vaccinated with homologous third-dose inactivated vaccine, so as to provide scientific basis for the optimization of the existing COVID-19 vaccine immunization strategy.

3.3.1 Pre-clinical immunogenicity evaluation (mouse model: intramuscular injection+mucosal immunization)

3.3.1.1 Specific IgG antibody test results

The results showed that Ad5-nCoV immunized by two routes showed good immunogenicity: after intramuscular injection, anti-S protein IgG antibody reached its peak at 28th day after vaccination, and then decreased slightly; IgG antibody in mucosal group reached its peak at 28 days after vaccination and remained stable for 56 days. In the high dose group, the IgG antibody titers of mucosal immunization group were higher than those of intramuscular injection group ($P < 0.0001$ at 42 days, $p=0.0001$ at 56 days), and there was no significant

difference between the two routes of administration in the middle and low dose groups at 42 days and 56 days after vaccination (p>0.05).

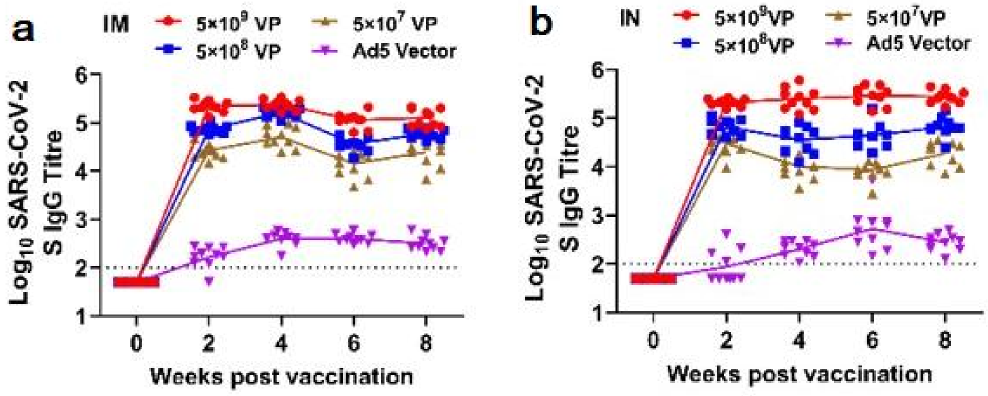

Fig. 3.3-1 Results of serum anti-S protein specific IgG antibody levels of mice on 14th, 28th, 42nd and 56th days after single immunization with different administration methods (a. intramuscular injection; B. mucosal immunity)

Fourteen days and 10 weeks after immunization, anti-S-protein specific IgG antibodies were detected in the bronchoalveolar lavage fluid of intramuscular injection and mucosal immunization group, but anti-S-protein specific IgA antibodies were only detected in mucosal immunization group, as shown in the following figure:

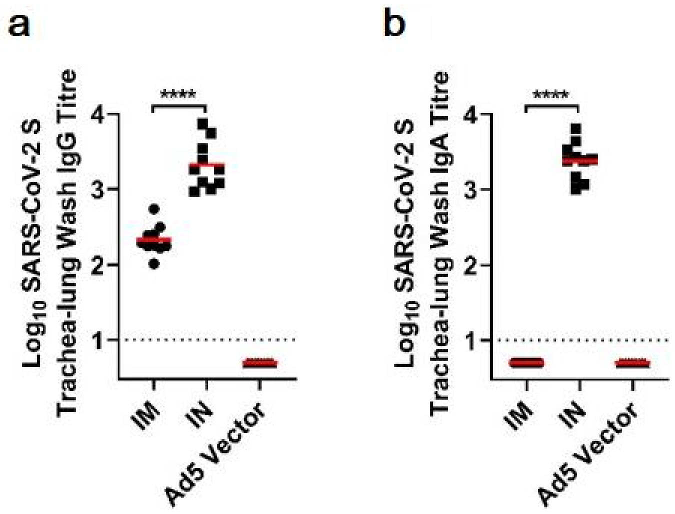

Fig. 3.3-2 IgG and IgA antibody levels in lung lavage fluid of mouse in middle dose group 14 days after single immunization with different administration modes
(a) IgG titer of lung lotion; B. IgA titer of lung lotion; IM: intramuscular injection; IN:

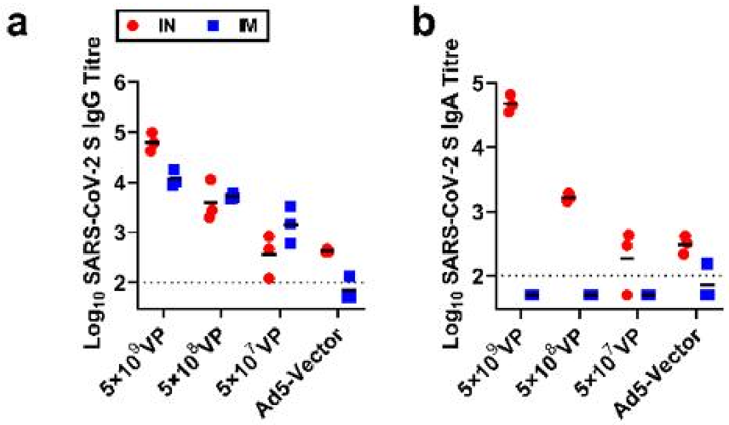

mucosal immunity)

Fig. 3.3-3 IgG and IgA antibody levels in mouse lung lavage fluid 10 weeks after single immunization with different administration methods

(a) IgG titer of lung lotion; B. IgA titer of lung lotion; IM: intramuscular injection; IN: mucosal immunity)

3.3.1.2 neutralizing antibody results

The neutralizing antibody (NAb) levels of anti-SARS-CoV-2 in mouse serum on 14th day, 28th day, 42nd day and 56th day after single intramuscular injection and mucosal immunization were detected by virus specific micro-neutralizing cytopathic test (see Figure 3.3-4 for details):

The results showed that Ad5-nCoV immunized by two routes showed good immunogenicity: the level of neutralizing antibody after mucosal immunization and intramuscular injection reached the peak at 42 days and 56 days after vaccination, respectively; The neutralizing antibody titers of mucosal immunization group in high dose group were significantly higher than those of intramuscular injection group at 28-56 days after vaccination ($P < 0.0001$ and $p=0.0021$ at 28-42 days, 56 days). There was no significant difference in neutralizing antibody titers between the two routes of administration in middle dose group at 42 days and 56 days after vaccination, and there was no significant difference in neutralizing antibody titers between the two routes of administration in low dose group at all time points after vaccination.

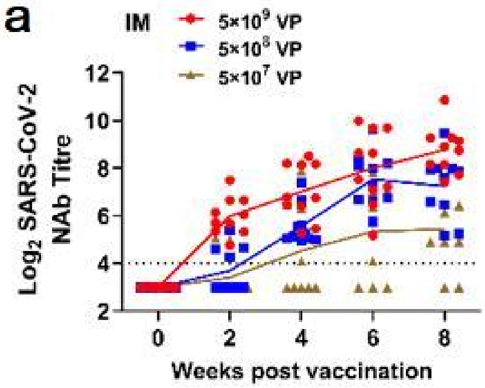

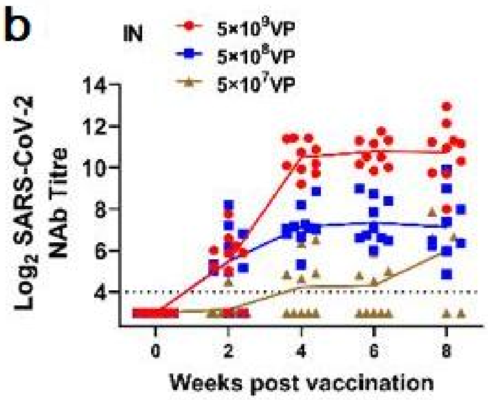

Fig. 3.3-4 Results of neutralizing antibody levels of anti-SARS-CoV-2 in mouse serum on 14th, 28th, 42nd and 56th days after single immunization with different administration methods (a. intramuscular injection; B. mucosal immunity)

Serum pseudovirus neutralizing antibody levels of mouse were detected on 14th, 28th, 42nd and 56th days after single intramuscular injection and mucosal immunization respectively (see Figure 3.3-5 for details). The results showed that the titer of neutralizing antibody of pseudovirus was equivalent to that of neutralizing antibody of real virus.

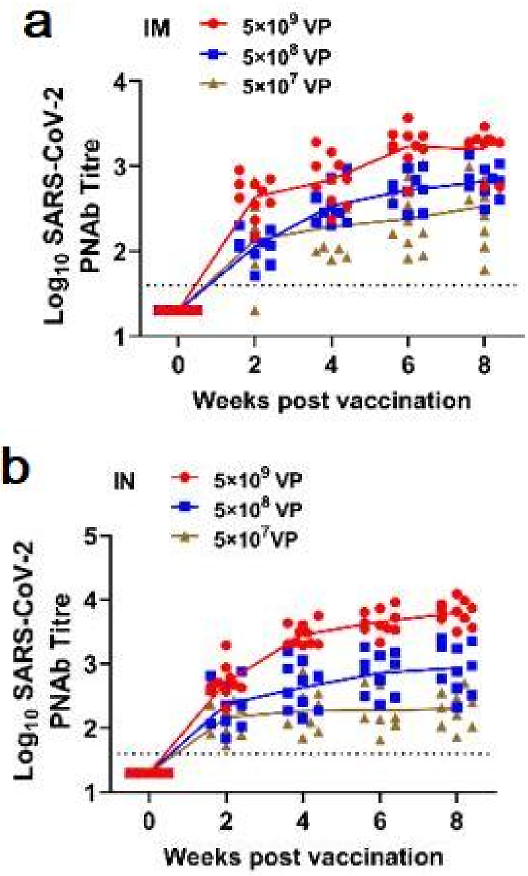

Fig. 3.3-5 Results of neutralizing antibody levels of pseudovirus in mouse on 14th, 28th, 42nd and 56th days after single immunization with different administration methods (a. intramuscular injection; B. mucosal immunity)

There is a good correlation between IgG antibody level, neutralizing antibody level and pseudovirus neutralizing antibody level at 42 days and 56 days after inoculation.

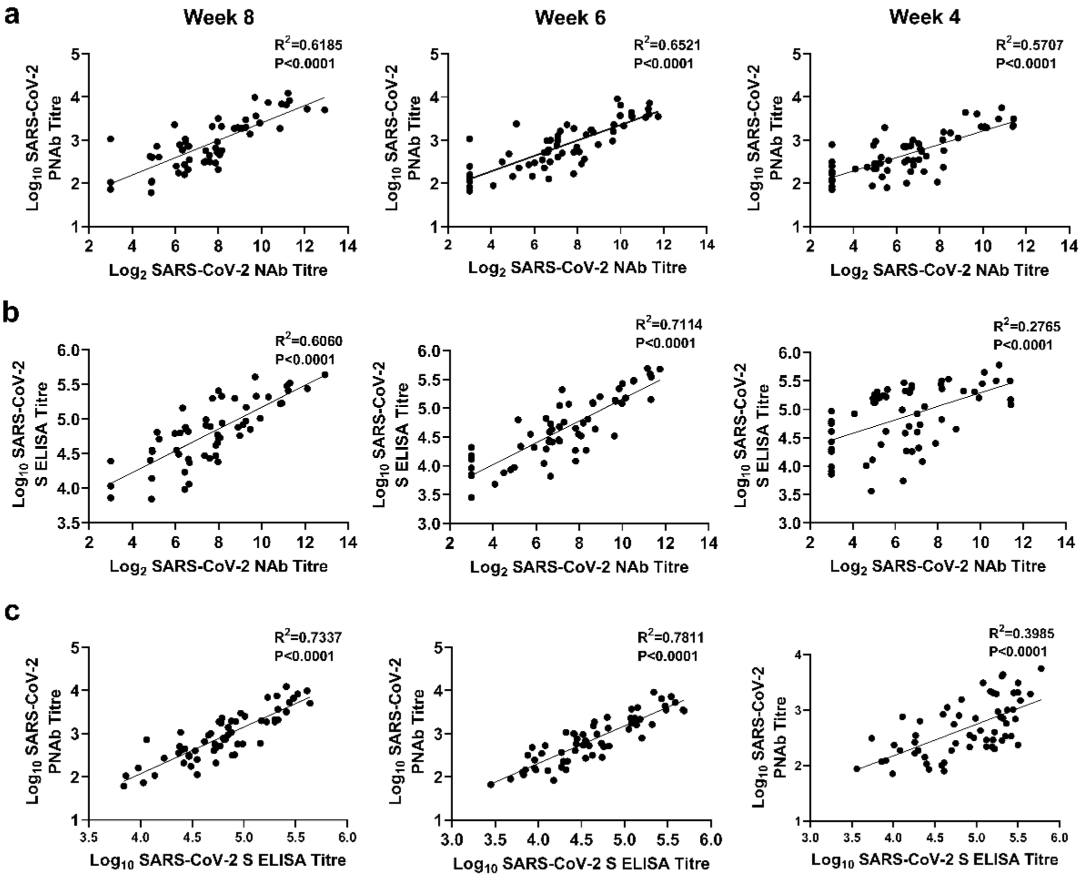

Fig. 3.3-6 Correlation between IgG antibody level, neutralizing antibody level and pseudovirus neutralizing antibody level in mouse immunized 3.3-6 Ad5-nCoV

3.3.1.3 Cellular immune response

Intracellular cytokine staining was used to detect the percentage of cytokine positive cells in CD8+ or CD4+T cells.

Both intramuscular injection and mucosal immunization in the middle dose group can significantly induce $CD8^+$and $CD4^+$T cells to produce IFNγ, TNFα and IL-2 on 14th day after immunization, and the intramuscular injection group is higher than the mucosal immunization group.

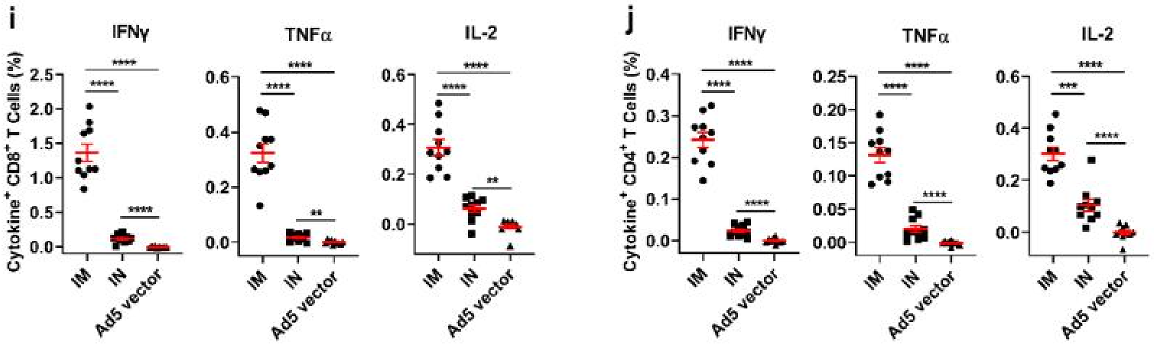

Fig. 3.3-6 Results of cellular immune response in mouse 14 days after single immunization

Ten weeks after immunization, the intramuscular injection group showed an increasing dose of cellular immune response, while the mucosal immunization group showed no obvious dose-dependent relationship.

3.3.2 Pre-clinical protective test

3.3.2.1 Ferret model (intramuscular injection+mucosal immunization)

3.3.2.1.1 Specific IgG antibody and neutralizing antibody results

18 ferrets were randomly selected and randomly divided into 3 groups: intramuscular injection, nasal drops and negative control with 6 ferrets in each group. All vaccinated ferrets produced anti-S protein specific IgG antibody and neutralizing antibody on 28th day after immunization, but none of them were detected in the control group. There was no significant difference between the two routes of administration.

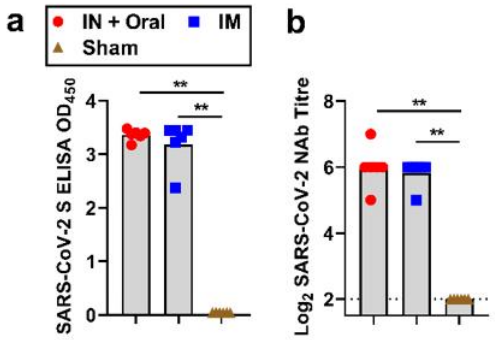

Figure 3.3-7 Results of IgG antibody and neutralizing antibody against S protein specificity of ferrets

3.3.2.1.2 Cellular immune response

Only 28 days after immunization, cellular immune responses were detected in 5 ferrets in IM group and 3 ferrets in mucosal immune group by IFNγ and ELISpot methods.

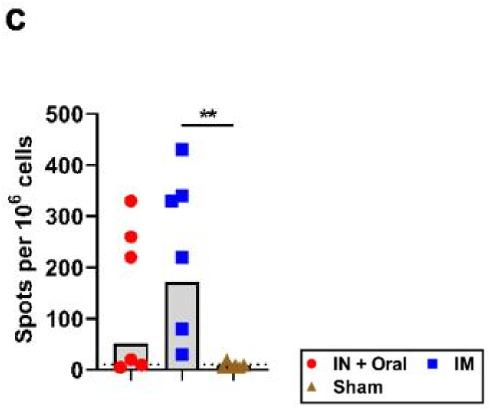

Figure 3.3-8 Results of cellular immune response of ferrets

3.3.2.2 Rhesus monkey conservation experiment (nebulized inhalation)

3.3.2.2.1 Immunogenicity of Different Doses

In the "Immunogenicity Study of Rhesus Monkeys with Different Doses and Different Needles" commissioned by Beijing Zhaoyan Company, the nebulized immunization is consistent with the clinical nebulized inhalation. Immunization doses were 1/2 human dose (2.5×10^{10}VP) and 3 human doses (15×10^{10}VP), respectively. nebulized immunization was carried out twice with an interval of two weeks. Blood samples were taken before and after each immunization to determine the antibody level in serum. ELISA was used to determine the antibody levels in the serum of the first and second immunizations, and JMP software was used for statistical analysis. The results showed that, for rhesus monkeys, the antibody level of 3HD after 1 and 2 injections of immunization was higher than 1/2HD.

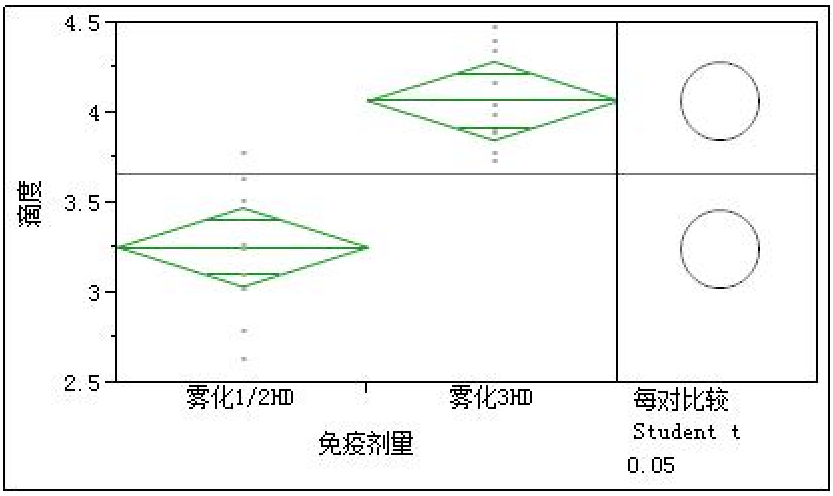

Fig. 3.3-9 titer of antibody in serum of different nebulized immunization doses 1

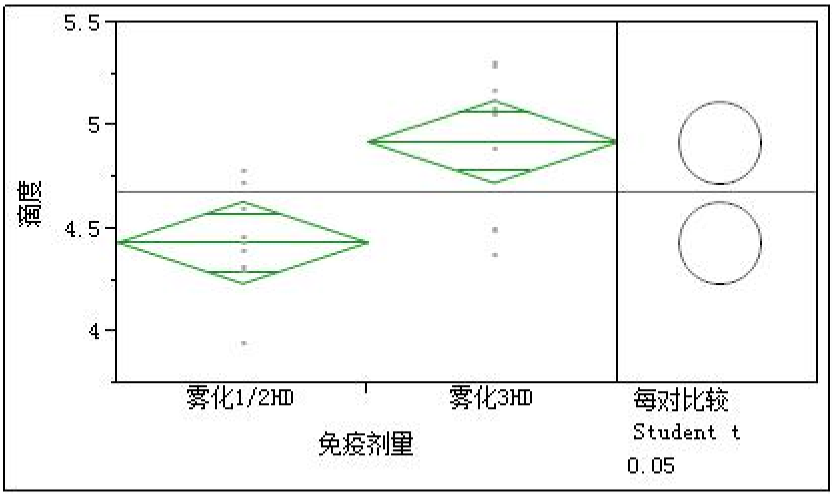

Fig. 3.3-10 Antibody titers in different nebulized immune doses 2 immune serum

3.3.2.2.1 Immunogenicity of different injections

Inhale the test sample for 1/2HD, immunize for three times, with an interval of two weeks. Before and after each immunization, take blood to determine the antibody level in serum. ELISA method was used to determine the antibody level in serum after the first, second and third immunizations. The results showed that the antibody level in the serum of the two atomized immunizations was significantly higher than that of the one, and the difference was statistically significant, but there was little difference between the three and two immunizations.

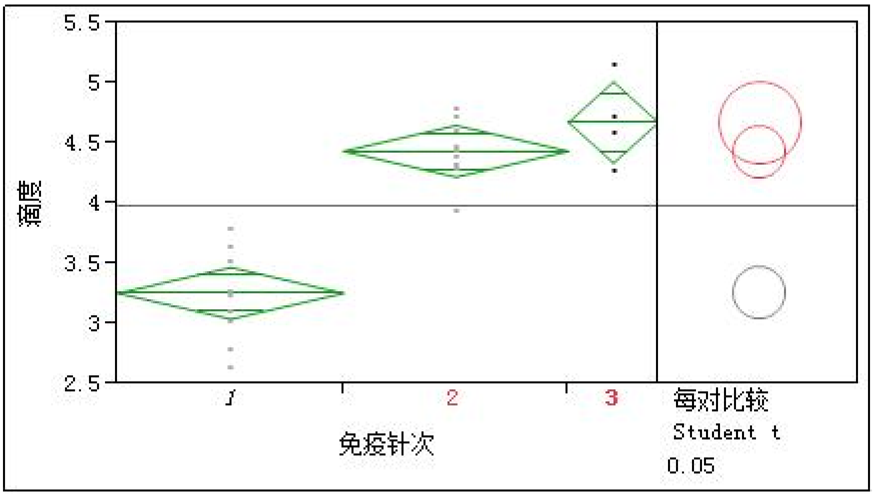

Fig. 3.3-11 Antibody Titers in Serum of Rhesus Monkeys at Different Immunization Times

3.3.3 Pre-clinical safety evaluation

3.3.3.1 Single-dose toxicity studies in SD mouses (mucosal immunity)

Under the experimental conditions, the recombinant COVID-19 vaccine (adenovirus type 5 vector) was given to SD mouses by intranasal instillation at a dose of 0.5×10^{11}vp/ animal (1 dose/animal) for many times. No toxic reaction was found, and the maximum tolerated dose (MTD) was greater than or equal to 0.5×10^{11}vp/ animal (1 dose/animal).

3.3.3.2 Toxicity studies of repeated administration of rhesus monkeys (mucosal immunity, nebulized inhalation)

Toxicity test of recombinant COVID-19 vaccine (adenovirus type 5 vector) after repeated nebulized inhalation or nasal spray to rhesus monkeys for 4 weeks and 2 weeks in recovery period;

During the experiment, no animals in each group were dead or dying, no abnormal reaction related to drug administration was observed clinically, and no allergic reaction symptoms were observed in animals after three times of drug administration. Compared with the same sex negative control group in the same period, The indexes of each test group of animals in different routes of administration include weight and weight gain, temperature, ophthalmological examination, clinical pathology (blood count, coagulation function, blood biochemistry, urine analysis), T lymphocyte subsets ($CD3^+$、 $CD3^+ CD4^+$、$CD3^+CD8^+$、$CD3^+CD4^+/CD3^+CD8^+$), serum cytokines (IL-2、IL-4、 IL-5、IL-6、TNF-α、IFN-γ)、C-reactive protein, serum complement (C3 、 C4), organ weight, viscera-body ratio, and viscera-brain ratio were not significantly changed or abnormal changes of toxicological significance. Before and after the first and last administration, there were no obvious abnormal changes or toxicological changes in the safety pharmacological indexes of animals in each group, including ECG waveforms and parameters, blood pressure (systolic blood pressure, diastolic blood pressure, mean arterial pressure) and respiratory function (respiratory frequency and tidal volume).

Immunogenicity test results showed that 10/10 of the animals in the negative control group were all negative for specific IgG antibody against S antigen, and only 1/10 of the animals could detect transient specific IgG antibody against adenovirus vector on D14. Two weeks after the first administration (D14), only a few animals produced weak specific IgG antibodies against adenovirus vector, with antibody titers ranging from 1: 100 to 1: 200. By the end of the recovery period (D43), there was no obvious change in the positive rate and titer of specific IgG antibody against adenovirus vector, and the antibody titer ranged from 1: 100 to 1: 400. Specific IgG antibodies against S antigen were produced in all animals in the low, high dose group by nebulized inhalation and high dose group by nasal spray at 2 weeks (D14) after the first dose, and the positive rate of antibody was 10/10. With the increase of administration times, the antibody titer range increased. Before the last administration (D28), the antibody titer range of nebulized inhalation group was 8721.886~201992.795, and that of nasal spray group was 579.049~10487.362. After two weeks of drug withdrawal, the antibody titers of each group did not decrease, ranging from 18154.480 to 227215.309 in the nebulized inhalation group to 1051.386 to 15979.091 in the nasal spray group.

The results of antinuclear antibody detection showed that during the experiment, no antinuclear antibody was detected in the negative control group and the animals in each test group with different routes of administration.

Pathological examination showed that there were no gross and histopathological changes related to the test products in all groups of animals at the end of drug administration (D32) and recovery period (D43). There was no irritating reaction in the local area after administration.

Conclusion: Under the experimental conditions, the recombinant COVID-19 vaccine (adenovirus type 5 vector) was given to rhesus monkeys by repeated nebulized inhalation at the dose of 2.5×10^{10}VP/ animal and 1.5×10^{11}VP/ animal, or repeated nasal spray at the dose of 1×10^{11}VP/ animal, once every two weeks, for a total of 3 times. During the trial, no toxic reactions were observed in each group of animals, that is, the No Observed Adverse Effect Level (NOAEL) were 1.5×10^{11} VP/unit and 1×10^{11} VP/unit, respectively. Two weeks after administration (D14), all animals produced strong specific IgG antibodies against S antigen, and the antibody titers increased with the increase of administration times. No immunotoxic reaction was found in animals. No irritating reaction was found locally after administration.

3.3.3.3 Study on repeated administration toxicity of cynomolgus monkeys (intramuscular injection)

Toxicity test of repeated intramuscular injection to cynomolgus monkeys for 2 weeks and recovery period for 2 weeks;

During the experiment, no animals in each group were dead or dying, no abnormal reaction related to drug administration was observed clinically, and no allergic reaction symptoms were observed in animals after two times of drug administration. During the experiment, compared with the same-sex negative control group in the same period, The indexes of low test product and high dose groups (1 dose/animal, 3 doses/animal) include body weight and weight gain, temperature, ECG waveform and parameters, blood pressure, ophthalmologic examination, clinical pathology (blood count, coagulation function, blood biochemistry, urine analysis), T lymphocyte subsets ($CD3^+$、 $CD4^+$、$CD8^+$、$CD4^+$/ $CD8^+$), Serum cytokines（IL-2、IL-4、IL-5、IL-6 、 TNF-α 、 IFN-γ ） , C-reactive protein, serum complement (C3, C4), organ weight, viscera-body ratio and viscera-brain ratio have no obvious changes or no toxicological abnormal changes.

3.4 Preliminary clinical research results

3.4.1 Sequential clinical study of muscle

On April 20, 2021, a single-center, randomized, observer-blind, parallel-controlled sequential immunization clinical trial was conducted in Lianshui County, Jiangsu Province, to evaluate the safety and immunogenicity of people aged 18-59 years who were given different doses of sequential booster vaccine.

The sample size is 300 people, and they are divided into 4 groups (sequential booster immunization group, routine booster immunization group, sequential immunization group and routine immunization group), with 100 people in the booster immunization group and 50 people in the routine immunization group. In the booster group, 3~6 months after the second inactivated vaccine immunization, sequential immunization was carried out; Routine immunization group received sequential immunization 1~2 months after the first dose of inactivated vaccine.

Preliminary safety results show that:

Booster immunization group: After the subjects finished two doses of inactivated vaccine, the incidence of adverse reactions and adverse events of the first dose of adenovirus vaccine was higher than that of the first dose of inactivated vaccine within 0-28 days, and the adverse reactions were mostly mild. No serious adverse events related to the vaccine occurred in the two groups.

Routine immunization group: After the subjects finished one dose of inactivated vaccine, the incidence of adverse reactions and adverse events in 0-28 days after the booster dose of adenovirus vaccine was higher than that in the booster dose of inactivated vaccine, and the adverse reactions were mostly mild. No vaccine-related serious adverse events occurred in the two groups during the trial.

Preliminary results of immunogenicity showed that:

Booster immunization group: in IgG typing, RBD antibody, neutralizing antibody against

eukaryotic virus and strain analysis, the experimental group with one dose of adenovirus vaccine was better than that with one dose of inactivated vaccine. The result of N protein antibody is opposite. The results of cell immunity showed that the positive rate of IL-5 and the immune response level of IL-5 and IL-13 cells were statistically significant ($P<0.05$) 14 days after the booster vaccination.

Routine immunization group: in IgG typing, RBD antibody, neutralizing antibody against eukaryotic virus and strain analysis, the booster dose of adenovirus vaccine is better than booster dose of inactivated vaccine; The result of N protein antibody is opposite. The results of cell immunity showed that only IL-5 was statistically significant between the two groups ($P<0.05$).

4. Introduction of vaccines

The recombinant COVID-19 vaccine (adenovirus type 5 vector) and the inactivated COVID-19 vaccine (Vero cell) are both one-injection immunization procedures, and the interval between vaccination and the third dose of inactivated vaccine is 6~9 months.

<u>Vaccine 1: recombinant COVID-19 vaccine (adenovirus type 5 vector) (hereinafter referred to as Ad5-nCOV).</u>

Production unit: Institute of Biological Engineering, Institute of Military Medicine, Academy of Military Sciences/Kangxinuo Bio-stock Company

Specification: 0.5ml/ bottle (5×10^{10}VP)

Doseage: 0.5ml(5×10^{10}VP) was injected into the lateral deltoid muscle of the upper arm.

Storage and transport conditions:

<u>Vaccine 2: inactivated COVID-19 vaccine (Vero cell) (hereinafter referred to as ICV)</u>

Production unit: Beijing Kexing Zhongwei Biotechnology Co., Ltd./Sinopharm Zhongsheng Beijing Biological Products Research Institute Co., Ltd.

Specification: 0.5ml/ bottle.

Dosage: 0.5ml

Approach: intramuscular injection of deltoid muscle in the lateral upper arm.

It should be stored and transported in the dark at 2~8°C to prevent freezing.

5. Test objective

5.1 Main purpose

To evaluate the immunogenicity and safety of a sequential booster dose of recombinant COVID-19 vaccine (adenovirus type 5 vector).

5.2 Secondary purpose

To evaluate the immunogenicity persistence of a sequential booster dose of recombinant COVID-19 vaccine (adenovirus type 5 vector).

6. Experiment design

Experimental design:

Randomized, blind and parallel controlled clinical study.

Random:

Subjects 6~9 months after 3 doses of inactivated vaccine were randomly assigned to the Ad5-nCoV group or the ICV group according to their age groups (18-59 years old and 60-80 years old) according to the ratio of 1:1. Among them, people aged 60-80 account for about 40%. An independent randomizing professional generates a random list of subjects through SAS 9.4 or above, and imports it into the interactive response technology (IRT) system, which can only be accessed by authorized personnel. Non-blind personnel of authorized research centers can obtain grouping information of subjects through IRT system, and use research vaccines of corresponding groups according to grouping information.

In this study, non-blind people were set up to distribute, configure and vaccinate. Non-blind people do not participate in other field work except distribution, configuration and vaccination, and other researchers remain blind.

Experimental design:

This project plans to enroll 200 subjects aged 18 and above. The subjects need to complete basic immunization with three doses of inactivated COVID-19 vaccine, and the interval between this booster immunization and the third dose of vaccination should be 6~9 months. Subjects aged 60 and above accounted for about 40%.

All subjects need to use diary card for systematic safety observation within 28 days after vaccination. All subjects need to complete the interview and blood sample collection on the day of vaccination, 14 days, 28 days, and 3 months after vaccination.

6.1 Study endpoint index

6.1.1 Main endpoint indicators

1) Incidence of Adverse Reactions (AR) in each group from 0 to 14 days after the boost immunization;
2) Geometric mean concentration (GMC)/geometric mean titer (GMT), seroconversion/seropositive rates, and geometric mean fold increase (GMFI) of antibodies that bind SARS-CoV-2-specific RBD and pseudovirus-neutralizing antibodies against Omicron BA.4/5 on day 14 post-booster dose.

6.1.2 Secondary endpoint indicators

Immunogenicity index:

Geometric mean concentration (GMC)/geometric mean titer (GMT), seroconversion/seropositive rates, and geometric mean fold increase (GMFI) of antibodies that bind SARS-CoV-2-specific RBD and pseudovirus-neutralizing antibodies against Omicron BA.4/5 on day 28 to 90 days after vaccination.

Safety indicators:

1) Incidence rate of adverse reactions/events in subjects from 0 to 28 days after the boost immunization;
2) Incidence of serious adverse events (SAE) in subjects within 28 days after the boost immunization.

6.1.3 Exploratory endpoint indicators

1) GMT, Influencing factors (including age, sex, BMI, and chronic diseases) to adverse reactions and immunogenicity.

6.2 Sample size estimation

(1) Hypothesis 1. The antibody level of 1,Ad5-nCoV group 14 days after booster immunization is not inferior to that of ICV booster immunization group;
(2) Hypothesis 2. The antibody level of 2,Ad5-nCoV group was better than that of ICV group 14 days after booster immunization.

6~9 months after vaccination with 3 doses inactivated vaccine ICV, people aged 60-80 accounted for about 40%. Subjects were randomly assigned to Ad5-nCoV group or ICV group according to 1:1. Ad5-nCoV is compared with ICV group. After hypothesis 1 is established, hypothesis 2 can be further statistically inferred. Because there are two end-point hypothesis tests in this project, the decision-making strategy of the multiplicity problem uses the fixed sequence method in the sequential strategy, and hypothesis tests are carried out in a predefined order. The nominal test level α of each hypothesis test is the same, that is, only when the original hypothesis is rejected in hypothesis test 1 will the next hypothesis test 2 be carried out, and the unilateral test level of both hypothesis tests is 0.025.

According to the reported research, inactivated vaccine boosters were performed 6 months after 3 doses of inactivated vaccine, and the antibody level after booster immunization was comparable to the peak antibody level after 3 doses of immunization. Therefore, it is estimated that the antibody level in ICV group will be 1: 150 (after 10 is the bottom log =2.18) after 14 days of immunization, assuming that the Ad5-nCoV can reach at least 1: 300 after sequential booster (after 10 is the bottom log = 2.48), the standard deviation is about 4 (fter 10 is the bottom log =0.6), the COV is estimated to be 2.415, and the sample size is calculated:

Hypothesis 1, the one-sided α of the inspection level = 0.025, the ratio of Ad5-nCoV group to ICV group is 1: 1, the non-inferiority limit of GMT ratio is 0.67 (after 10 is the bottom log =-0.174), and the actual GMT ratio of the two groups is 2.00. When the sample size of each group is 80 people, there are 160 people in both groups, the calculated power of the test is 99.86%, which is in line with.

Hypothesis 2, the one-sided α of the inspection level = 0.025, and the ratio of the two groups is 1:1, when the sample size of each group is 80, the total number of the two groups is 160, and the GMT level of the Ad5-nCoV-IM group after immunization is better than that of the ICV group, the estimated power of the test is 88.16%, which meets the test requirements.

To sum up, considering that the two hypotheses are met, the shedding rate of the subjects in the experiment (20.00%) and the random block group, the sample size of the Ad5-nCoV-IM group and the ICV group are set to be 100 people each, with a total of 200 people in both groups. The sample size of subjects in each group according to age distribution is arranged in Table 2 below.

Table 2 Design of each group and sample size

Test grouping	Age group (years)	group	Sample size

			(example)
Ad5-nCoV	18~59	A1	60
	60~80	A2	40
ICV	18~59	B1	60
	60-80	B2	40
total			200

6.3 Numbering Rules

6.3.1 Screening number

After the subjects arrive at the scene for registration, a unique screening number is given, and each screening number corresponds to one subject. Number rule of screening number for each site: all are S+ 3 digits.

6.3.2 Random numbering rules

Before vaccination after the first visit to the group, the field researchers assigned a random number to the subjects through IWRS according to their group order. Each subject has a unique random number, and each random number corresponds to the only subject until the end of the study.

This project plans to enroll 200 subjects aged 18 and above. In this study, the subjects who completed three doses of inactivated COVID-19 vaccine were randomly assigned to receive Ad5-nCoV or ICV at the ratio of 1:1. Random numbering rule: A+ 3 digits are numbered continuously. Subjects who fall off without vaccination after randomization can be substituted for randomization. For example, if the subject A+001 falls off without vaccination, the next enrolled subject is a substitute subject with a random number of A+1001. If the substitute subject falls off without vaccination, the next enrolled subject is the second substitute subject with a random number of A+2001, with a maximum of 9 substitute subjects.

6.4 Blind vaccine method

This clinical trial is designed by blind method, that is, neither researchers nor subjects know the grouping of the study.

6.4.1 Packaging of vaccine for test

In this study, central randomization system (IWRS) was used for randomization, and independent non-blind personnel were set up to check and prepare the drugs, and then the vaccinators were vaccinated, so the vaccine was not packaged blindly.

6.4.2 Editing blindness

In this study, central randomization system was used for randomization, and no blinding was done. Independent non-blind people were assigned to the corresponding groups according to the subjects displayed by the randomized system, and the vaccines of the corresponding groups were checked and then submitted to the preparers for preparation after re-checking, and then the vaccinators completed the vaccination. Non-blind people sign confidentiality agreements, and only participate in vaccine access, vaccine preparation and vaccine packaging management, and do not participate in other research links.

6.4.3 Blind bottom preservation

Subjects' randomization table: A blind randomizer applied SAS software (version 9.4 or above) and adopted the block randomization method, setting the sample size, the ratio between groups (1:1), the block length, the number of blocks and the number of seeds to generate the blind base of random numbers. The blind bottom will be saved in IWRS in electronic form, which will be used to expose the blind after the database is locked. Until the end of the study (except for emergency blinding conditions), the information of all subjects' grouping and the information of experimental medication can not be made public.

6.4.4 Blindness Maintenance

This trial is a single-center, randomized, double-blind clinical trial. During the whole clinical trial, the subjects, researchers, data analysts and the sponsor all maintained blindness, and adopted a number of measures to maintain blindness.

Non-blind people sign confidentiality agreements, and only participate in random, vaccine access, vaccine preparation and vaccine packaging management. They are not allowed to disclose the grouping contents of subjects to other staff members who participate in clinical research. No one who has the opportunity to participate in on-site research is allowed to contact or ask about the contents of the subjects' groups.

6.4.5 Blindness Removal

According to the research progress, the time of blinding will be decided by the researcher. After the last subject completed V4 (the 3th month), the research data were sorted out, the blind audit and database locking were completed, and the main researcher initiated the application for uncovering the blind in IWRS with the joint witness of all research parties. After uncovering the blind, the electronic version of the blind was delivered to the statistician for statistical analysis.

6.4.6 Emergency blind uncovering

The emergency blinding of this trial will be applied for and completed through IWRS. If serious complications and adverse events occur during the trial, which will affect the choice of treatment measures, the researcher thinks that it is necessary to know the group of the subjects, so that it can be done urgently. When emergency blindness breaking is needed, the researcher or the person authorized by the researcher logs in IWRS through the account password to complete emergency blindness breaking and reveal the grouping information of the subject. Researchers should record the reasons for emergency blindness removal in IWRS. Inform the main research unit, ethics office and clinical inspector in time after blindness.

6.5 Test period

Estimated start time: May 2022.

Estimated end time: December 2022.

6.6 Suspension and early termination of research

The researchers collected the adverse events of the subjects after vaccination. In case of violation of the program, GCP requirements, ethical requirements, etc., the main researcher, ethics committee or administrative department have the right to suspend or terminate the research, and have the obligation to inform other parties and subjects and explain the reasons.

In case of any of the following circumstances, the trial will be suspended, and the researcher

shall hold an expert group meeting to decide whether to terminate the clinical trial in advance:

- −There was 1 case of grade 4 or above adverse reaction possibly related to vaccination;
- −1 case of serious or life-threatening SUSAR occurred;
- −The number of subjects with adverse reactions with severity of Grade 3 and symptoms lasting for > 48 hours and not relieved to Grade 1 or 2 exceeded 15% of the vaccinated subjects;

One of the following situations occurs, the study is terminated:

- −The ethics committee requires the complete termination of the experiment and explains the reasons;
- −The administrative department requires a complete termination of the test and explains the reasons.

7. Subjects

7.1 Selection of subjects

Based on the principle of informed consent and voluntary participation, volunteers aged 18 and above were selected to complete the basic immunization with three doses of inactivated Covid-19 vaccines, and the interval between this booster immunization and the third vaccination should be 6~9 months.

7.2 Inclusion criteria

1) When screening volunteers aged 18 and above;
2) Get the informed consent of volunteers and sign the informed consent form;
3) Volunteers are able and willing to comply with the requirements of clinical trial protocol and can complete 6-month follow-up study;
4) Three doses of inactivated Covid-19 vaccines have been vaccinated, and the interval between this vaccination and this vaccination was 6~9 months.

7.3 Exclusion criteria

1) Those who have a medical history or family history of convulsion, epilepsy, encephalopathy and psychosis;
2) Those who are allergic to any component of the research vaccine, and those who have severe vaccine allergic reaction, allergic history or asthma history in the past;
3) Serious adverse events related to vaccination occurred after vaccination with COVID-19 vaccine in the past;
4) Women with positive urine pregnancy test or breast-feeding;
5) People with acute febrile diseases, infectious diseases and a history of SARS;
6) Underarm temperature > 37.0℃;
7) Suffering from serious cardiovascular diseases, such as arrhythmia, conduction block, myocardial infarction, severe hypertension and uncontrollable drugs (systolic blood pressure ≥180mmHg and/or diastolic blood pressure ≥110mmHg when measured on site), etc.;
8) Suffering from severe chronic diseases, such as diabetes, thyroid diseases, etc., which are in the progressive stage and cannot be controlled smoothly;

9) Congenital acquired angioedema/neuroedema;
10) One year before receiving the experimental vaccine, he had urticaria;
11) Have asplenia or functional asplenia;
12) Suffering from pulmonary dysfunction such as chronic obstructive pulmonary disease and pulmonary fibrosis;
13) Have a history of Covid-19 infection/illness;
14) In the past 21 days, he has been to medium and high-risk areas or has a history of leaving the country, and has a history of epidemiological contact in COVID-19;
15) The researcher's judgment based on various medical, psychological, social or other conditions may be contrary to the experimental plan or affect the informed consent of the subjects.

7.4 Criteria for Subjects to Terminate the Test Early

Early termination means that the subject did not attend the end visit stipulated in the protocol, did not conduct the research steps, did not conduct the follow-up and did not collect more information about the subject since the last visit.

1) the subject requests to withdraw from the clinical trial;

2) Those who leave the observation area and can't complete the test process are regarded as voluntarily withdrawing from the test;

3) The subject's health condition does not allow him to continue to participate in this experiment;

4) Any other reasons considered by the researcher.

7.5 Rejection criteria

Each visit should be measured by the following criteria. If the following conditions exist, the subjects do not need to withdraw from the study, but the possibility of being analyzed as a group meeting the program set will be affected.

1) Does not meet the selection criteria;

2) meet the exclusion criteria;

3) Those who fail to follow up the data and information after vaccination;

4) after randomization, the information and data are seriously missing;

5) The subject has not been vaccinated, received the wrong vaccination or the incorrect dose.

8. Methods and procedures

8.1 Selection of subjects

The recruitment of clinical trials will be conducted at the research site, and the recruitment time will be about 2 weeks before the start of visit 1(V1). The recruitment of subjects is carried out by researchers with working experience.

8.2 Informed consent

When obtaining and recording informed consent, researchers should abide by relevant

regulations, the ethical principles stipulated in GCP and Helsinki Declaration. Before the start of the research, the researcher should get the written approval/consent of the ethics review committee on the informed consent and other documents provided to the subjects.

Before participating in this clinical study, researchers should tell volunteers and witnesses about the contents of the informed consent form, and give volunteers/witnesses enough time to consult the details of the study before they sign the informed consent form. When explaining the information of informed consent to many people, each volunteer/witness should be given the opportunity to ask the researcher questions individually before signing the informed consent form.

The researcher must keep the informed consent signed by each volunteer for the inspection by the drug administration department and regulatory administrators. At the same time, a copy of the signed informed consent form with name and date should be provided to the subject.

Subjects must obtain their informed consent.

8.3 On-site registration

After the volunteers have informed consent and signed the informed consent form, the researchers will assign the screening numbers according to the order of arrival. The screening number is S+3 consecutive numbers.

8.4 Physical examination and screening

Subjects need to have a general physical examination, including height/weight/temperature, blood pressure, and urine pregnancy test for women of childbearing age (not yet menopausal).

According to the "inclusion and exclusion criteria", the consulting doctor inquired about the medical history and screened the patients. Only those who passed the screening can join the group and participate in randomization.

8.5 Subjects were randomly divided into groups.

In the random semicolon group, researchers assigned unique random numbers in turn through IWRS system according to the order of arrival of the subjects, and could not skip or leave the numbers.

8.6 Vaccine and use

Before vaccination, the information of the subjects should be checked, and vaccination can only be carried out if it meets the requirements of this clinical research plan. Emergency medicines such as epinephrine hydrochloride and other emergency equipment such as simple ventilator and ECG monitor should be available at the vaccination site.

8.6.1 Research vaccine

Ad5-nCoV is jointly developed by Institute of Bioengineering, Institute of Military Medicine, Academy of Military Sciences, and Concino Biotech Co., Ltd. ICV vaccine was produced by Beijing Kexing Zhongwei Biotechnology Co., Ltd./Sinopharm Zhongsheng Beijing Biological Products Research Institute Co., Ltd

8.6.2 Immunization routes and immunization procedures

Subjects were injected with one dose of research vaccine intramuscularly at 1(V1) time point according to one dose immunization program. The vaccine should be shaken thoroughly before use. Cracks in the penicillin bottle, unclear labels or failure, and abnormal appearance of the vaccine should not be used. In intramuscular injection group, the vaccine was injected intramuscularly at the attachment of deltoid muscle on the lateral upper arm. It is recommended that the maximum range of syringe used for inoculation is 1mL, and the minimum scale is 0.01mL.

8.6.3 Storage and transportation of vaccines for research

1) Vaccine packaging: blind packaging is not required in this study.

2) Storage of vaccines: vaccines for research must be stored in a safe and locked place, which cannot be contacted by unauthorized persons. The temperature of vaccine storage place should be controlled in the range of 2~8℃; The storage temperature of vaccine should be recorded once in the morning and afternoon of each working day.

3) Vaccine transportation: the A5-NCOV vaccine manufacturer is responsible for transporting A5-NCOV from the place of production to each clinical trial site unit and cold storage, and submitting the transportation temperature record (in line with the cold chain temperature of the vaccine) and the inspection report (qualified) of the experimental vaccine to the researcher together with the experimental vaccine, which will be checked and signed by the vaccine managers of each clinical trial site unit. ICV vaccine is provided in clinical trial site.

The vaccine is transported from the research site unit to the inoculation site, from the inoculation site to the research site unit, and the refrigerator or freezer where the experimental vaccine is stored at the inoculation site. Each cold chain device is equipped with a thermometer, and the vaccine administrator records the temperature every 30 minutes, filling in the transportation and preservation temperature records in detail. During transportation, the storage temperature (2~8℃) must be ensured, any overtemperature must be reported to the research party for instructions, and all vaccine transportation processes must be recorded.

8.6.4 Combined medication

If a medical incident occurs during the study period, corresponding treatment and medical treatment are allowed, but the drug used or medical treatment should be recorded in time. No matter what kind of vaccine the subjects were vaccinated during the study period, detailed records were required.

The subjects' combined medication should be recorded in the diary card within 28 days after each dose of vaccination, and the combined medication of SAE should be recorded in EDC system during the whole observation period.

Subjects are not advised to take other vaccines during the study period, except those who need emergency vaccination due to emergency events, including rabies vaccine, tetanus vaccine, or other vaccines that need emergency vaccination. All the combined vaccines should be recorded in the diary card within 28 days after vaccination.

8.6.5 Organization and management of research vaccines

Researchers will be responsible for vaccination, recovery and inventory of research

vaccines, and record and archive them accurately and timely. After the end of the study, the researcher must return all the remaining vaccines and outer packages for research to the researcher. Researchers should not use research vaccines on any occasion or under any circumstances other than those specified in this research program.

8.7 Follow-up and evaluation of safety

8.7.1 Safety observation

All subjects should observe at the vaccination site for 30 minutes after vaccination. Researchers should systematically observe each subject, record the local and systemic reactions of the subjects within 30 minutes and record the severity.

All subjects were followed up for safety within 14 days after vaccination. Researchers should systematically observe the adverse reactions of the subjects, and the subjects should fill in diary cards according to their own symptoms and signs. From the 15th day to the 28th day after vaccination, the adverse events after vaccination were observed by the method of active reporting and regular follow-up. The researcher inquired and checked the diary card, and instructed the subjects to improve the content. From 28 days to 3 months after vaccination, the safety of the subjects was followed up by active reporting.

8.7.2 Contents and indicators of safety observation

Based on the types and incidence of adverse reactions observed in previous clinical trials, and referring to the adverse reactions listed in the instructions of similar products on the market, this clinical trial is mainly based on the Guiding Principles of Classification Standards of Adverse Events in Clinical Trials of Preventive Vaccines (NMPA [2019] No.102). See Table 8.7-1-8.7-2 for related safety observation contents and index grades after vaccination, and Table 8.7-3 for safety grades of other adverse events.

8.7-1 Grading table of adverse events at intramuscular vaccination sites (local)

Symptoms/signs	Grade 1	Grade 2	Grade 3	Grade 4
Pain	Do not affect or slightly affect physical activity.	Affect physical activity	Affect daily life	Loss of basic self-care ability, or hospitalization
Induration *, swelling * * #	Diameter 2.5~ < 5cm or area 6.25~ < 25 cm^2 and does not affect or slightly affect daily life	Diameter 5~ < 10 cm or area 25~ < 100 cm^2 or affect daily life	Diameter ≥ 10 cm or area ≥ 100 cm^2 or ulceration or secondary infection or phlebitis or aseptic abscess or wound drainage or seriously affect daily life	Abscess, exfoliative dermatitis, , dermal or deep tissue necros
Rash *, blush *	Diameter 2.5~	Diameter 5~ <	Diameter ≥ 10 cm or	Abscess,

* #	< 5 cm or area 6.25~25cm^2 and does not Affect or slightly affect daily life	10 cm or area 25~ < 100 cm^2 or affect daily life	area≥ 100 cm^2 or ulceration or secondary infection or phlebitis or aseptic abscess or wound drainage or serious affect daily life	exfoliative dermatitis, dermal or deep tissue necrosis
Itch	Itching at the vaccination site, relieved by itself or within 48 hours after treat	Itching at the vaccination site, which does not resolve within 48 hours after treatment	Affect daily life	NA
Cellulitis	NA	Non-injectable treatment is required (e.g. oral antibacterial, antifungal, antiviral therapy)	Intravenous treatment is required (e.g. intravenous antibacterial, antifungal, antiviral therapy)	Sepsis, or tissue necrosis, etc.

Note: *: in addition to directly measuring the diameter for grading and evaluation, the progress of the measurement results should also be recorded.

** the maximum measuring diameter or area should be used.

the evaluation and grading of induration and swelling, rash and redness should be based on the functional level and the actual measurement results, and the indicators with higher classification shouldbe selected.

Table 8.7-2 Non-vaccination site (systemic) adverse event grading table (sore throat is systemic adverse event in intramuscular injection)

Symptoms /signs	Grade 1	Grade 2	Grade 3	Grade 4
Diarrhea	Mild or transient, 3~4 times a day, abnormal stool, or mild diarrhea last less than 1 week.	Moderate or persistence, 5~7 times a day, abnormal stool characteristics, or diarrhea > 1 week.	> 7 times/day, abnormal stool, or hemorrhagic diarrhea, orthostatic hypotension, electrolyte imbalance, requiring intravenous infusion > 2L.	Hypotension shock, hospitalization.

Symptoms /signs	Grade 1	Grade 2	Grade 3	Grade 4
Nausea	Transient (< 24 hours) or intermittent and food intake is normal.	Continued nausea leads to reduced food intake (24-48 hours)	Persistent nausea results in almost no food intake (> 48 hours) or requires intravenous fluid replacement	Life-threatening (eg hypotension shock)
Anorexia	Loss of appetite, but not reduced food intake	Loss of appetite and reduced food intake, but no significant weight loss.	Loss of appetite and weight loss.	Need for intervention (e.g. gastric tube feeding, parenteral nutrition)
Vomiting	1~2 times /24 hours and dose not affect the activity.	3~5 times /24 hours or activity is restricted	> 6 times/24 hours or need intravenous rehydration	Hypotension shock requires hospitalization or other means of nutrition
Fatigue	Does not affect daily activities.	Affect normal daily activities.	Seriously affect daily activities and can't work.	Emergency or hospitalization
Headache	Does not affect daily activities and requires no treatment.	Transient, slightly affects daily activities, and may require treatment or intervention.	Seriously affects daily activities and requires treatment or intervention.	Intractable and requires emergency or hospitalization
Arthralgia	Mild pain, without hindering function.	Moderate pain; need analgesics and/or pain that impedes function, but does not affect daily activities.	Severe pain; need analgesics and/or pain affecting daily activities	Disability pain
Chest pain	Mild pain, does not hindering function.	Moderate pain; need analgesics and/or pain that impedes function, but does not affect daily activities.	Severe pain; need analgesics and/or pain affecting daily activities	Disability pain
Pharyngal gia	Transient, without treatment, does not affect daily activities.	Sore throat slightly affects daily activities.	Severe sore throat, which seriously affects daily activities, requires medical treatment.	NA
Runny	Transient,	Persistent runny nose,	Treatment is	Emergency or

Symptoms /signs	Grade 1	Grade 2	Grade 3	Grade 4
nose	without treatment	effective treatment	uncontrollable.	hospitalization
Sneeze	Transient, without treatment	Continuous sneezing, effective treatment.	Treatment is uncontrollable.	Emergency or hospitalization
Cough	Transient, without treatment	Continuous cough, effective treatment	Treatment is uncontrollable.	Emergency or hospitalization
Non-injection-site muscle pain	Does not affect daily activities.	Slightly affect daily activities	Severe muscle pain, seriously affecting daily activities	Emergency or hospitalization
Itching at non-inoculation site (No skin damage)	Slight itching, not affecting or slightly affecting daily life.	Itching affects daily life.	Itching makes it impossible to carry out daily life.	NA
Abnormal skin mucosa	Erythema/itching/color change	Diffuse rash/maculopapules/dryness/desquamation	Blister/exudation/desquamation/ulcer	Exfoliative dermatitis involving mucosa, or erythema multiforme, or suspected Stevens-Johnsons syndrome.
Acute 、allergic reaction **	Local urticaria (blister) without treatment	Local urticaria, requiring treatment or mild angioedema, without treatment.	Extensive urticaria or angioedema requiring treatment or mild bronchospasm.	Anaphylactic shock or life-threatening bronchospasm or laryngeal edema
Syncope	Close to syncope without losing consciousness (pre-syncope)	Lose consciousness, but do not need treatment.	Loss of consciousness, requiring treatment or hospitalization.	NA
Constipation *	Need stool softener and diet	Need laxative drugs	Stubborn constipation needs manual dredging or	Toxic megacolon or intestinal

Symptoms /signs	Grade 1	Grade 2	Grade 3	Grade 4
	adjustment.		enema.	obstruction
Dysphagia	Mild discomfort when swallowing	Diet restriction	Diet and conversation are very limited; you can't eat solid food	Can't eat liquid food; Need parenteral nutrition
Arthritis	Mild pain with inflammation, erythema, or swelling of joints;but does not interfere with function	Moderate pain with inflammation, erythema, or swelling of joints; impairs function but does not affect daily activities	Severe pain with inflammation, erythema, or joint swelling; affecting daily activities	Permanent and/or disabling joint injury
New convulsion	NA	NA	1~3 convulsions	Long-term and repeated convulsions (e.g. status epilepticus) or difficult to control (e.g. intractable epilepsy)
Acute bronchospasm	Transitivity; Without treatment; FEV_1% is 70%~80%	Need treatment; Bronchiectant therapy returned to normal; FEV_1% is 50%~70%	Bronchiectant treatment can not return to normal; FEV_1% is 25%~50% or the intercostal depression persists.	Cyanosis; FEV_1%<25%; Or need intubation.
Dyspnea	Dyspnea during exercise	Dyspnea during normal activities	Difficulty breathing at rest	Dyspnea, requiring oxygen therapy, hospitalization or assisted breathing.
Insomnia *	Mild difficulty in falling asleep, not affecting or slightly	Moderate difficulty in falling asleep, affecting daily life	Serious difficulty in falling asleep, seriously affecting daily life, requiring treatment or	NA

Symptoms /signs	Grade 1	Grade 2	Grade 3	Grade 4
	affecting daily life.		hospitalization	
Irritate or restrain	Mild irritation or mild inhibition	Irritable or lethargic	Unable to soothe or respond poorly	NA
Mental disorders (including anxiety, depression , mania and insanity) should report detailed symptoms	Mild symptoms, no need for medical treatment or behavior does not affect or slightly affect daily life	Have clinical symptoms, need to see a doctor or behavior affects daily life	Need hospitalization or inability to support daily life	Have a tendency to harm oneself or others or acute insanity or loss of basic self-care ability
Non-injection-site pain# (Specify the location when reporting)	Minor pain that does not affect or slightly affect daily lif	Pain affects daily life	Pain can't carry on daily life	Disability pain, loss of basic self-care ability
Fever ※ [axillary temperature (℃)]	37.3 ~ < 38.0	38.0 ~ < 38.5	38.5 ~ < 39.5	≥ 39.5, last more than 3 days

Note: FEV_1% refers to forced expiratory volume in one second (FEV_1)/forced vital capacity (FVC)

*For constipation and insomnia, attention should be paid to the changes before and after vaccination.

**It refers to type I hypersensitivity reaction.

#It refers to pain in other non vaccination sites except muscle pain, joint pain and headache.

※ The axillary temperature is usually adopted in China, which can be converted into oral and anal temperature when necessary.Generally, mouth temperature=axillary temperature+0.2 ℃;

Anus temperature=axillary temperature+(0.3~0.5 ℃).When persistent high fever occurs, the cause of high fever should be determined as soon as possible.

Local adverse events are all solicited events. Among the above non vaccination site (systemic) adverse events, pharyngodynia (pharyngodynia is a systemic adverse event in intramuscular injection), fever, diarrhea, fatigue, nausea, anorexia, vomiting, headache, cough, arthralgia, chest pain, non vaccination site myalgia, non vaccination site pruritus (no skin damage), skin and mucous membrane abnormalities, runny nose, sneezing is a solicited adverse event, and the rest are non solicited adverse events.For other non solicited adverse events, please refer to the investigator's manual.

The adverse events that do not reach grade 1 in the above table can be recorded as level 0.

General principles for classification of other adverse events:

For clinical abnormalities not involved in the above grading table, the intensity grading assessment of adverse events shall be carried out according to the following standards:

Table 8.7-3 Classification of Other Adverse Events

Grade 1	Grade 2	Grade 3	Grade 4	Grade 5
Mild: Short-term (< 48 hours) or mild discomfort, no influence on activities, treatment not indicated	Moderate: Mild or moderate restricted activities, presentation indicated possibly, treatment not indicated or mild treatment indicated	Severe: Significant restricted activities, presentation and treatment indicated, hospitalization indicated possibly	Critical: Life-threatening possibly, severely restricted activities, intensive care indicated	Death

8.7.3 Outcome of AEs

The outcomes of Ars/AEs include: 1) Recovery; 2) Recovered but sequelae; 3) Improvement; 4) Continuity; 5) Death; 6) Loss of visit.

8.7.4 Relationship between AEs and vaccination

The researchers should try their best to explain the AE and evaluate its possible causal relationship, that is, the causal relationship between the vaccination and alternative causes (such as the medical history of basic diseases, combined treatment).This applies to all AEs, both serious and non serious.

Causality evaluation will be determined by the extent to which the event can be reasonably explained in one or more of the following aspects:

- In the past, similar reactions have been observed for such preparations;
- For similar types of preparations, the same events have been reported in the literature;
- The event occurred with the vaccination of the study vaccine in time, and reappeared after the re vaccination of the study vaccine.

According to the definition, all collected AEs (that is, all collected reported local adverse events) at the vaccination site will be considered to be related to vaccination.The causality of the AE should be evaluated by the researcher according to the following questions. According to your judgment, whether there is a reasonable possibility that the AE is caused by vaccination:

Absolutely unrelated: adverse events are caused by other factors, and there is sufficient evidence to prove that adverse reactions/events are caused by other reasons and not related to vaccination.

Possibly unrelated: the occurrence of adverse events may be caused by other factors, such as the clinical condition of the subject, other treatments or concomitant drugs, which are inconsistent with the known adverse reactions of vaccination.

It may be relevant: the adverse events are consistent with the known experimental vaccine information, have a reasonable time sequence with vaccination, and/or have occurred adverse events for vaccination.It has a causal relationship with the experimental vaccine, but may also be related to other factors.

Affirmative: the adverse event is consistent with the known information of the test vaccine, and has a causal relationship with the test vaccine, and this relationship cannot be explained by other factors, such as the clinical condition of the subject, other treatments or concomitant drugs.In addition, adverse events recurred when subjects used the trial vaccine again.

Unable to judge: according to the existing information, the researcher believes that it is impossible to judge, and no further follow-up information can be obtained.

In this test, the events that are sure to be relevant, may be relevant and cannot be judged will be statistically analyzed as relevant.

8.7.5 Treatment of Adverse Reactions/Events

Adverse events: refer to all adverse medical events that occur after the subjects are vaccinated, which can be manifested as symptoms and signs, diseases or abnormal laboratory tests, but may not have a causal relationship with the vaccine/vaccination.

Adverse reaction: refers to any harmful or unexpected reaction in clinical trials that may be related to vaccination.

Serious adverse events refers to the following important medical events whether related to clinical trials of vaccines or not, including: 1) death; 2) Life-threatening; 3) resulting in prolonged hospitalization or hospitalization time; 4) resulting in permanent or significant disability/loss of function; 5) Congenital abnormality or birth defect; 6) Other important medical events, such as those listed above may occur without treatment.

SUSAR: Susar: Susar refers to the harmful reaction of the subject at any dose, which has nothing to do with the purpose of medication. After analysis, it is considered that the relationship with the drug is at least possibly related; Unexpected refers to the nature, degree, consequence or frequency of adverse reactions, which is different from the expected risks described in previous plans or other relevant materials (such as researcher's manuals and instructions).

Subjects should report any clinically significant diseases/events to researchers as soon as possible after vaccination. Researchers should follow up the adverse events/serious adverse events until the symptoms disappear or stabilize. All drug treatment, medical treatment and disease outcome will be recorded at each follow-up.

In case of serious adverse events/reaction, the researcher should take necessary measures quickly, fill in the serious adverse events Report Form within 24 hours, and report it to the main researcher and vaccine manufacturer by fax or E-mail.

8.7.6 serious adverse events's reporting procedure

Any serious adverse events, whether it is related to the experimental vaccine or not, the SAE reporter at the research site must fill in the serious adverse events Report Form within 24 hours after being informed, and report it to the main researcher and vaccine manufacturer (pv@cansinotech.com) by fax or E-mail, including the description, occurrence time and type,

duration, causal relationship with vaccination, results and treatment methods (symptomatic treatment) of adverse events.

After receiving the report of serious adverse events, the researcher shall, together with the researcher, comprehensively consider the duration, scope, intensity and outcome of adverse events and the wishes of the subjects to decide whether the subjects will continue to participate in the trial or terminate the trial ahead of time. The vaccine manufacturer shall judge whether the SAE is a suspicious and unexpected serious adverse reaction (SUSAR). If so, the vaccine manufacturer shall promptly report to National Medical Products Administration Drug Evaluation Center and the main researcher, who will report the suspicious and unexpected serious adverse reaction to the ethics committee.

For suspicious and unexpected serious adverse reactions (SUSAR) that are fatal or life-threatening, vaccine manufacturers should report them to National Medical Products Administration Drug Evaluation Center as soon as possible, but not more than 7 natural days, and report them within the next 8 days to improve the follow-up information.

For the non-lethal or life-threatening suspicious and unexpected serious adverse reactions, or other potentially serious safety risks, vaccine manufacturers should report to National Medical Products Administration Drug Evaluation Center as soon as possible, but within 15 natural days. Researchers should truthfully record the serious adverse events, and evaluate and discuss it in the final report after the completion or termination of the experiment.

Table 8.7-4 Contact information and reporting time limit of SAE report

Main researcher: Huakun-Lu	E-mail:hklv@cdc.zj.cn	Time limit: within 24 hours after learning SAE
Zhejiang Provincial Center for Disease Control and Prevention Ethics Review Committee	E-mail:zgjiang@cdc.zj.cn	Time limit: within 24 hours after learning SUSAR
Vaccine manufacturer	E-mail:PV@cansinotech.com	Time limit: within 24 hours after learning SAE

8.7.7 Treatment of pregnancy events

Pregnant women are not allowed to participate in the vaccination of this test vaccine.

Before vaccination, urine pregnancy test was conducted on the subjects, and those with positive urine pregnancy test were not allowed to join the group. If a pregnancy event occurs within 3 months after vaccination, the pregnancy event reporter at the research site should fill in the Pregnancy Report Form within 5 natural days after being informed, and report it to the main researcher and vaccine manufacturer (pv@cansinotech.com and the ethics committee by fax or E-mail. Pregnancy events should be followed up until the end of pregnancy or 6 weeks after the baby is born.

8.8 Evaluation of immunogenicity

8.8.1 Specimen collection

10.0ml of venous blood was collected on day 0 (V1), day 14 (V2, window period +3 days), day 28 (V3, window period +5 days) and month 3 (V4, window period +30 days) after

vaccination in each group.

Use a vacuum blood collection tube containing serum isolate, and do not refrigerate the sample before centrifugation, so as to minimize the risk of hemolysis and avoid blood cell contamination when transferring the serum to the target serum tube. Blood samples were collected on the same day, and serum antibodies were measured and stored at -20℃ or below.

The blood sample of this clinical trial will be used to evaluate the immune response level of the vaccine. If it is used in other studies, it needs the approval of the ethics committee and the consent of the subjects. Except the serum for inspection, the remaining backup samples are transferred to a third-party laboratory after the clinical end. Blood samples will not go abroad or leave the country.

8.8.2 ELISA detection of S-RBD IgG antibody against SARS-CoV-2

Serum anti-SARS-CoV-2 S-RBD IgG antibody levels were detected before booster immunization and 14 days, 28 days, and 3 months after immunization.

8.8.3 VOC/VOI cross neutralization detection

The levels of VOC/COI cross-neutralizing antibodies were detected before booster immunization and 14 days after immunization.

Calculation method of uncertainty value: When calculating GMT, GMI and positive conversion of antibodies determined by ELISA and neutralization test, if the initial dilution is negative, it is calculated as half value of the initial value, and if it is greater than the highest dilution, it is calculated as the highest dilution.

8.8.4 Serum number

1) the numbering rules of serum tubes for anti-SARS-CoV-2 specific neutralizing antibody detection: research number-visit number -1;

2) The numbering rules of serum tubes for ELISA detection of anti-SARS-CoV-2 S-RBD IgG antibody: research number-visit number -2; 1

3) Numbering rules of backup serum tubes: research number-visit number -3 and research number-visit number -4.

Serum minimum 0.8ml per tube

8.8.5 Preservation and transportation of specimens

Unified operation standards shall be adopted in the process of storage and transportation. The storage temperature should be below -20℃, and it should be transported to the testing laboratory in time.

8.9 Site Work Flow

8.9.1 Overview of visiting process

The subjects completed all the research contents through five visits from the screening before entering the group to the end of all the research. See Table 8.9-1 for the specific time, time window and interview contents of each visit.

Table 8.9-1 Visiting Process

Visit No.	V1	V2	V3	V4
Visiting time	D-7-D0	D14	D28	The 3th month

Time window	0 days	+3 days	+5 days	+30 days
informed consent	●			
Registration/verification of identity information	●	●	●	●
Physical examination (blood pressure, height, weight, temperature)	●			
Urinary pregnancy (women who have not yet reached menopause)	●			
Medical history inquiry and admission screening	●			
Random number assignment	●			
blood specimen collection	10ml	10ml	10ml	10ml
Vaccination[a]	●			
Distribution diary card (within 14 days)	●			
Distribution of diary card (after 14 days)		●		
Audit diary card		●	●	
Safety observation[b]	●	●	●	
SAE observation and report[c]	●	●	●	●

a) If the researcher thinks that the health condition of the subjects on the day of vaccination is temporarily unsuitable for vaccination, the vaccination can be delayed within one week;

b) Observe and record all adverse events from day 0 to day 28 after vaccination, whether related to vaccine or not;

c) The researcher shall fill in the "serious adverse events Report Form" to complete the first report within 24 hours after learning that the subject has SAE, and complete the summary report after the event;

d) If the screening period is divided into two days, blood pressure and temperature must be re-measured on the day of enrollment.

Table 8.9-2 Sampling Schedule of Clinical Trial Visit

Number of visits	V1	V2	V3	V4
Visiting time	D0	D14	D28	The 3th month
Immunogenicity	10ml	10ml	10ml	10ml

8.9.2 Field personnel grouping and task division

Table 8.9-3 Field Grouping and Task Division of Clinical Trials

serial	Group	Duty	visit

number			
1)	Informed consent group	Get the informed consent of the subject.	V1
2)	Registration group	And verification of registration identity information.	V1~V4
3)	physical examination	Have a general physical examination before joining the group.	V1
4)	Urine test group	Urine was collected for urine pregnancy test.	V1
5)	Screening group	Ask about the selection and exclusion criteria and fill in the screening form.	V1
6)	Research number assignment group	Assign random number	V1
7)	Immunogenic blood sampling group	Collect 10.0ml venous blood.	V1~V4
8)	Vaccination group	Vaccination is carried out according to the procedure and route.	V1
9)	Liuzu	Observe the vaccinated subjects for at least 30 minutes.	V1
10)	Journal card recovery group	Recycle the diary card, and review the standardization and accuracy of the diary card.	V2，V4

8.9.3 Site management

Table 8.9-4 Site Management Groups and Responsibilities

serial number	group	duty	visit
1)	Medical guidance group	① Maintain the order;<br>② When necessary, lead the subjects to each inspection team to complete the items examined by the subjects one by one.	V1~V4
2)	Serum separation and management group	① Receive the blood samples delivered on site and fill in the handover form;<br>② Separating serum and subpackaging into EP tube;<br>③ Preservation and regular transportation of biological specimens;<br>④ Record the type, quantity and specimen number of transported specimens.	V1~V4
3)	Vaccine management group	① Receiving and storage of vaccines;<br>② Record of cold chain temperature;<br>③Fill in vaccine distribution and recovery records;	V1

		④ Check the quantity when closing seedlings.	
4)	Safety observation group	① Routine safety follow-up;<br>② Complete serious adverse events survey;<br>③ Report serious adverse events in time;	V1~V4
5)	File management group	① Field data recovery and management;<br>② File entry;<br>③ Data archiving.	V1~V4
6)	Logistics support group	① Material support;<br>② Vehicle support.	V1~V4
7)	Emergency rescue team	①Emergency security incidents, professional ambulance personnel on-site emergency treatment;<br>② If the situation is serious, rush to the hospital.	V1
8)	Quality control group	① Check before the project starts;<br>② The subjects were examined within one week after joining the group;<br>③Routine inspection of the process;<br>④ On-site shutdown inspection.	V1~V4

8.10 Data management

In this study, EDC system is used to collect and manage research data, and the system keeps a complete revision track to ensure the authenticity, completeness and accuracy of clinical trial data. The data management process should conform to GCP specifications to ensure the traceability of clinical trial data.

8.10.1 Design and Establishment of Database

The project database (eCRF) is established by the database designer, and CDISC standard is adopted as far as possible.

After the database is established and tested, the authorized personnel PI, Sub-I, CRC, PM, CRA, DM, etc. of each role can be officially put into use after training.

The data administrator writes a data management plan (DMP), which should be finalized before the screening of the first subject.

8.10.2 Data entry

The researcher or the person authorized by the researcher completes the online data entry in time after the interview.

Researchers need to approve the data in eCRF to verify that the data recorded in eCRF is true. After the data entry is completed, any data changes need to be comments and will be automatically recorded in the system.

8.10.3 Audit of data records

The auditor shall regularly or irregularly audit the data records entered into EDC to ensure that all the entered data are consistent with the original documents. If there is any inconsistency, the auditor needs to send a query to the researcher at the corresponding place in the EDC system, and the researcher needs to verify the original data and update the entered content until the EDC system is completed. Before locking the library, the auditor should carefully verify the original data of the subjects and the necessary signatures of the researchers.

8.10.4 Data verification

According to the data verification plan (DVP), the data management personnel query and manage the test data.

When data is entered into EDC system, if there is any illogical data, the system will automatically check and Query; These queries need to be reviewed and answered by researchers or authorized personnel. When the updated data makes the logical check untenable, the Query will automatically close; When the query is closed automatically, DM can audit. When the problem is not solved, DM can manually add questions and continue to communicate with the research site until the problem is solved.

In addition to the automatic verification by the system, when the researchers need to clarify/verify/confirm the questions checked manually by SAS programming or data administrator, they can manually add Query to the EDC system.

Before locking the database, the data administrator needs to ensure that all Query are cleaned up, and the researcher completes the electronic signature on the EDC system. So as to ensure the completeness and accuracy of patient data.

8.10.5 Medical Coding

Medical coders carry out medical coding work. Medical coding of non-solicited adverse events. Adverse events will be coded according to Meddra (version 21.1 or above) dictionary.

In the process of coding, DM can query researchers online in real time if any medical terms cannot be coded due to improper, inaccurate and vague provision.

Before locking the database, medical codes need to be medically audited.

8.10.6 Database Locking

Complete the list of database locks. According to the procedures of database locks, data managers, statistical analysts, clinical inspectors' representatives, researchers' representatives, etc. sign a written approval document for database locks, and the data manager will export it to the database in the specified format and submit it to statisticians for statistical analysis. After the data is locked, if there is definite evidence to prove that it is necessary to unlock it, researchers and relevant personnel should sign the unlocking document.

8.10.7 External data management

Immunogenicity data is managed as external data. For data transmission requirements, please refer to the External Data Transmission Protocol. Data management reviews and consistently checks external data.

8.10.8 eCRF archiving

At the end of the trial, each patient's eCRF was exported to PDF for electronic archiving, and the CD-ROM was burned and stored in the clinical trial unit.

8.11 Statistical analysis

8.11.1 Statistical Analysis Plan

The statistician (the third party) undertakes the task of statistical analysis, and participates in the whole process from experimental design, implementation to analysis and summary. After the experimental scheme is approved by the ethics committee, the researcher and the statistician will coordinate to establish a database and formulate a statistical analysis plan, which will be submitted to the main researchers, biostatisticians and data administrators for discussion and

approval, so as to determine the following contents: the selection of analysis data sets, data statistics methods, etc.

8.11.2 Research hypothesis

（1） Hypothesis 1: The antibody level of Ad5-nCoV group 14 days after booster immunization is not inferior to that of ICV booster immunization group;

Non-inferiority test is carried out, and the test assumptions are as follows:

H_0(invalid hypothesis): T/C $\leq$ 0.67

H_1(alternative hypothesis): T/C > 0.67

（2） Hypothesis 2: The antibody level of Ad5-nCoV group 14 days after booster immunization is better than that of ICV booster immunization group.

The test hypothesis is as follows:

H_0(invalid hypothesis): T/C $\leq$ 1

H_1(alternative hypothesis): T/C > 1

T = Test group: GMT of virus neutralizing antibody level at 14 days after the booster immunization of Ad5-nCoV group;

C = Control group: GMT of virus neutralizing antibody level in ICV group 28 days after booster immunization. α is 0.025 on one side (0.05 on both sides).

When the lower limit of one-sided 97.5% confidence interval of GMT ratio ($GMT_{3\text{-pin ICV+Ad5-nCoV}}/GMT_{3\text{-pin ICV+ICV}}$) of virus neutralizing antibody is greater than 0.67 after 14 days of vaccination of two populations, it is considered that the non-inferiority effect is established.

The lower limit of one-sided 97.5% confidence interval of GMT ratio ($GMT_{3\text{-pin ICV+Ad5-nCoV}}/GMT_{3\text{-pin ICV+ICV}}$) of virus neutralizing antibody was greater than 1 after 14 days of vaccination in two populations. It was considered that the Ad5-nCoV group was superior to the ICV group.

8.11.3 Selection of analysis datasets

Safety Evaluation Dataset (SS): All subjects who are randomized and vaccinated should be evaluated for safety. Data that violates the scheme should not be rejected.

Immunogenicity evaluation dataset:

Full Analytical Dataset (FAS):FAS is an ideal population determined according to ITT (Intention to treat) principle. All the enrolled and vaccinated subjects are included in FAS set, regardless of whether their baseline antibody level is positive or not, regardless of whether they seriously violate the experimental protocol.

Per-protocol Datase (PPS): it is a subset of FAS. The subjects in this data set are more compliant with the protocol, have no significant violation of the experimental protocol during the study period, and all the subjects who are eligible for inclusion but do not meet the exclusion criteria, have been randomized into groups, completed immunization, completed the immunogenicity evaluation before immunization and at the corresponding time point, and have the antibody test results. .

Per-protocol Datase (PPS2): it is a subset of PPS. The subjects in this data set are more compliant with the protocol, have no major violation of the experimental protocol during the

study period, and all the subjects who meet the inclusion/exclusion criteria and have 6-month immunogenicity results. PPS2 will be used for immune persistence analysis. PPS2 population will be defined in SAP. In this experiment, PPS was used as the main analysis set. At the same time, however, FAS should be analyzed, and any inconsistency between FAS and PPS analysis results should be discussed in the report.

8.11.4 Data statistics method

Unless otherwise stated, all statistical tests will use a two-sided test with α=0.05, and the confidence interval will use a two-sided 95% confidence interval (one-sided confidence interval is 97.5%). Summarize the continuity variables with descriptive statistics, including the number of cases, average, median, standard deviation, maximum and minimum. The description of the classification index uses the number and percentage of all kinds of examples.

The statistics will be further explained in the Statistical Analysis Plan (SAP), and SAP will finalize it before the database is locked.

8.11.4.1 Demographic data and baseline characteristics

Descriptive statistics were used to summarize the demographic data and baseline characteristics of each group and the total.

Demographic data include: enrollment and interview completion (screening failure, program violation); Number of people in each group, age and gender distribution.

Baseline features include: distribution of antibodies in each group and before total immunization.

8.11.4.2 Safety analysis

Adverse events will be coded by MedDRA dictionary. Analysis of adverse events will be based on adverse events (TEAE) after vaccination. TEAE is defined as an adverse event that occurs during vaccination or worsens during vaccination compared with before vaccination. The incidence of AE will be described according to System Organ Classification (SOC) and Preferred Terminology (PT). At the same time, provide a similar summary and list of SAE and adverse events leading to research interruption.

8.11.4.2.1 Incidence of adverse reactions 30 minutes after vaccination

The number, number and percentage of adverse reactions in each study group 30 minutes after vaccination were counted. According to the severity and symptoms, count the number, number and percentage of adverse reactions/adverse events in each study group 30 minutes after vaccination. Chi-square test or Fisher exact probability method was used for group comparison.

8.11.4.2.2 Incidence of adverse reactions 0~14 days after vaccination

The number, number and percentage of adverse reactions in each study group from 0 to 14 days after vaccination were counted. According to the severity and symptoms, the number, number and percentage of adverse reactions in each study group from 0 to 14 days after vaccination were counted. Chi-square test or Fisher exact probability method was used for group comparison.

8.11.4.2.3 Incidence of adverse events from 0 to 28 days after vaccination

The number, number and percentage of adverse events in each study group from 0 to 28

days after vaccination were counted. According to the severity and symptoms, the number, number and percentage of adverse events in each study group from 0 to 28 days after vaccination were counted. Chi-square test or Fisher exact probability method was used for group comparison.

8.11.4.2.4 SAE within 28 days after vaccination

The number, number and percentage of SAE cases in each study group within 28 days after vaccination were counted. According to the severity and symptoms, the number, number and percentage of SAE cases in each study group within 28 days after vaccination were counted. Chi-square test or Fisher exact probability method was used for group comparison.

8.11.4.3 Immunogenicity analysis

8.11.4.3.1 The Geometric Mean Titers (GMT), seroconversion rate and Geometric Mean of the fold Increase (GMI) of the specific neutralizing antibody against SARS-CoV-2 of the subjects after 14 days, 28 days, and 3 months of booster immunization.

The Geometric Mean Titers (GMT) of each visit in each study group were counted, and described by the number of cases, geometric mean, standard deviation, median, quartile, maximum, minimum and 95%CI. T-test was used to compare the differences between groups. Draw a reverse cumulative distribution map. Stratified by age.

The Geometric Mean of the fold Increase (GMI) of each visit of each research group was counted, and described by the number of cases, geometric mean, standard deviation, median, quartile, maximum, minimum, 95%CI, etc. T-test was used to compare the differences between groups. Stratified by age.

The seroconversion rate of each visit in each research group was counted, expressed by the number and percentage of cases, and 95%CI of positive conversion rate was calculated. Chi-square test or Fisher exact probability method was used for comparison between groups. Stratified by age.

8.11.4.3.2 Geometric Mean Concentration (GMC), Geometric Mean of the fold Increase (GMI) and seroconversion rate of anti-SARS-COV-2 S-RBD IgG antibody after 14 days, 28 days, and 3 months of booster immunization.

The Geometric Mean Concentration (GMC) of each visit of each research group was counted, and described by the number of cases, geometric mean, standard deviation, median, quartile, maximum, minimum and 95%CI. T-test was used to compare the differences between groups. Draw a reverse cumulative distribution map. Stratified by age.

The Geometric Mean of the fold Increase (GMI) of each visit of each research group was counted, and described by the number of cases, geometric mean, standard deviation, median, quartile, maximum, minimum, 95%CI, etc. T-test was used to compare the differences between groups. Stratified by age.

The seroconversion rate of each visit in each research group was counted, expressed by the number and percentage of cases, and 95%CI of positive conversion rate was calculated. Chi-square test or Fisher exact probability method was used for comparison between groups. Stratified by age.

8.11.4.3.3 The geometric mean titer (GMT), positive conversion rate and geometric mean growth factor (GMI) of the cross neutralization of some VOC/VOI of the subjects after 14 days of booster immunization.

The geometric mean titers (GMT) of each visit in each study group were counted, and described by the number of cases, geometric mean, standard deviation, median, quartile, maximum and minimum. T-test was used to compare the differences between groups. Draw a reverse cumulative distribution map. Stratified by age.

The geometric average growth multiple (GMI) of each visit of each research group was counted, and described by the number of cases, geometric mean, standard deviation, median, quartile, maximum, minimum, 95%CI, etc. T-test was used to compare the differences between groups. Stratified by age.

The seroconversion rate of each visit in each research group was counted, expressed by the number and percentage of cases, and 95%CI of positive conversion rate was calculated. Chi-square test or Fisher exact probability method was used for comparison between groups. Stratified by age.

See the Statistical Analysis Plan (SAP) for detailed statistical analysis methods.

8.11.5 Analysis arrangement

The first stage analysis:

After 14 days, the results of safety and immunogenicity data were verified, and the first stage analysis was carried out.

Final analysis:

The final analysis was carried out after the results of safety data and immunogenicity data for 3 months were verified.

8.11.6 Analysis software

This test adopts SAS 9.4 or above software for analysis.

9 Monitoring of clinical trials

9.1 Responsibilities of each party

9.1.1 Researchers

The main researcher should manage and clearly divide the work of all the people involved in clinical trials.

Researchers should keep the personal data of the subjects confidential. The original record book, eCRF form or other documents should be identified only by the tested code and random number. The researcher keeps the identification list and screening registration form (including complete name, age and address) of the subjects in the researcher's file. According to the principle of GCP, the original data of each subject are allowed to be monitored, checked and audited.

9.1.2 Inspector

Monitor (CRA) shall conduct on-site follow-up inspection according to a certain time rule. During the audit, it is necessary to check whether the original data are consistent with the

information in eCRF, that is, its accuracy and completion. If the CRF is found to be inconsistent with the original data, it is necessary to urge the researchers to revise it. Inspectors will evaluate the process of informed consent, transportation and storage of experimental vaccines, experimental documents and experimental progress. The inspector should check the compliance of the researcher with the scheme (or the scheme amendment), observe the test procedure and discuss some problems with the researcher. There should be an audit log to record the on-site audit.

9.2 Personnel training

The training targets include field operators, laboratory operators and clinical medical staff. The training includes the relevant contents of this research, research process, clinical techniques, emergency response methods, etc.

9.2.1 Field operators

All participants in this clinical trial are researchers, and they are required to have medical (nursing) professional qualification certificates (including the qualification certificates of practicing doctors, employment certificates of medical technicians, etc.). On-site operators should provide resumes (signed with date and name) and relevant documents. These materials are kept in the training documents by the person in charge. Participants need to register in the training registration form. A brief description of each training topic or task, including references to relevant documents, version number and training date should be filled in the training meeting record form. If new personnel are assigned to new positions, the person in charge should provide training according to the specific situation. The training content should be related to employees' (new) posts, especially for atomization inhalation operators, on-site practical operation training and practical training are required.

9.2.2 Laboratory operators

Laboratory operators should be qualified as technicians or above. Training in GCP, scheme and standard operating procedures is required, and the laboratory operation can only be carried out with the authorization of the main researcher after passing the examination.

The evaluation contents include:

1) quality of work;

2) mastery of new technologies and procedures;

3) Whether it can comply with relevant technical instructions and procedures;

4) Whether the results can be properly interpreted;

5) Make no mistakes;

6) The test results meet the acceptance criteria defined in relevant documents;

When new or modified experimental processes or techniques are to be implemented, training must be increased.

9.2.3 Clinical medical staff

Doctors are required to have doctor's qualification certificate or doctor's practice certificate, nurses are required to have nurse's practice certificate, and experimental technicians are required to have employment certificate.

Emergency doctors and nurses should have professional qualifications, cardiopulmonary

resuscitation and other skills, and are currently engaged in emergency work. They are familiar with the emergency treatment methods of common adverse reactions of vaccination, especially the emergency treatment of immediate hypersensitivity, and the green channel process of emergency treatment.

The main researcher or CRA etc. will provide unified training to the medical staff involved in each functional link, and the training contents refer to GCP and Field Operation Manual, etc.

9.2.4 Auditor (CRA)

According to the Measures for the Administration of Drug Registration, in the process of drug clinical trials, the applicant shall appoint personnel with certain professional knowledge to supervise the implementation of the Quality Management Standards for Clinical Trials of Vaccines (for Trial Implementation). Auditors involved in this study should be familiar with the following:

1) Good Manufacturing Practices for Clinical Trials of Drugs (GCP) and various laws and regulations;

2) Technical Guidelines for Clinical Trials of Vaccines (for Trial Implementation);

3) Clinical trial plan;

4) Informed consent;

5) vaccine storage, transportation, distribution and use;

6) Vaccination field use form and filling requirements.

9.3 Experimental reagents and methods (specimen testing unit)

9.3.1 Reagent Management

1) using standardized inspection methods;

2) Conduct irregular quality monitoring;

3) The reagent should be stored at the specified temperature, and a temperature monitoring system should be provided to monitor the preservation of the reagent. Cabinets and rooms for storing reagents stored at room temperature should be locked;

4) After the reagent arrives, its expiration date should be recorded in the reagent management log;

5) All stored reagents should be checked regularly for expiration. Expired reagents shall be treated according to corresponding regulations;

6) When the reagent is opened for use, the initials of the user's name, the starting time and the expiration time should be indicated on the bottle body.

9.3.2 Experimental Methods

According to all kinds of specimen testing procedures.

9.4 vaccine management

The experimental vaccine should be managed by a special person, and no one can contact it without the permission of the main researcher. The temperature of vaccine transportation and storage should be controlled within 2~8℃. The researcher is responsible for regularly exporting the temperature records of the cold storage for experimental vaccines. If there is any temperature deviation, that is, the temperature exceeds the specified range of 2~8℃, report it to the researcher.

Establish a work form for vaccine handover, registration, use and recovery, fill it out as required, and keep it in the work record. Ensure the quality of experimental vaccines and prevent unlisted vaccines from entering the market.

1) Record of vaccine handover: Cornino Bio-Co., Ltd. provides the experimental vaccine and the vaccine handover form. When the researcher receives the vaccine, it is necessary to verify whether the vaccine quantity, packaging are complete and cold chain system instructions are normal. Sign the handover form between both parties.

2) Record of vaccine registration and use: the researcher shall establish a record of vaccine registration and use, and record in detail the number of vaccinations, the remaining number or the loss number every day.

3) Record of vaccine recovery: the researcher will return the remaining experimental vaccines and the Vaccine Handover Sheet.

4) At the end of the project, the vaccine can be destroyed or returned to the manufacturer on the spot with the approval of the manufacturer. The main researcher has the responsibility to explain any difference in the number of vaccines.

9.5 Site and laboratory supervision

The on-site auditor is responsible for supervising the whole process of clinical trials, ensuring that the clinical trials meet the requirements of GCP and clinical trial scheme, and are completed within expectations.

1) supervise the storage, transportation, distribution, use and destruction of the remaining vaccines;

2) Participate in the selection of subjects, specimen collection, vaccination and side effect observation, laboratory testing, statistical analysis of results and collation of clinical reports;

3) Regularly arrive at the research site for supervision and timely (within 3 days) submit a written supervision report to the main researcher of the applicant and the unit in charge of clinical trials, including the whole process of clinical trials;

4) Deviations in clinical trials shall be settled by the inspectors and researchers through consultation, and major events shall be reported to the ethics committee.

9.6 Quality control of documents and materials

9.6.1 Original data

On-the-spot documents of 9.6.1.1 researchers

All on-site documents and materials shall be classified and stored according to data types. The data types are as follows (adjusted according to the situation of the research site):

1) original record book;

2) Informed consent;

3) Record of specimen collection;

4) vaccine immunization records;

5) Cold chain records;

6) Records of vaccine handover, use, distribution and recovery;

9.6.1.2 Laboratory Documents

1) Specimen test results and records (test report)

The process includes the acceptance of the specimen to the final test result report, including the following documents:

A) Transportation: specimen transportation list, specimen transportation problem log and specimen invalidation record form;

B) Specimen tracking: internal circulation table of specimens;

C) Specimen detection: serum antibody detection;

2) Statistical analysis data of specimen test results

The original data of the test results are entered into Excel file by the laboratory staff, which is saved in the computer of the laboratory. The second laboratory staff reviewed the input data again. When the review data entry is completed, the Excel file will be transferred to the data administrator.

9.6.2 Case Report Form (eCRF)

Researchers will transcribe two copies of eCRF for each subject. One eCRF is provided to the researcher, and the second one is kept by the researcher. Only researchers and approved staff are allowed to visit eCRF during the trial.

ECRF is used to record the data of clinical trials and is an important part of clinical trials and research reports.

No matter whether the subject finishes or withdraws from the experiment, the researcher must sign the eCRF to declare that the recorded data is accurate. For the subjects who terminate the trial, the reason for termination should be recorded on their eCRF.

ECRF should reflect the situation of the subjects in each stage of the experiment. The name of the subject cannot appear on eCRF, and the appropriate code and initials of the subject must be used.

All data on eCRF are from the original data and are consistent with the original data. All data recorded in eCRF shall be recorded in the original data.

Researchers and other relevant personnel should provide written documents about research exchanges, meetings and any modifications to the scheme, and all documents agreed by both parties should be made in duplicate, which should be kept for the record.

9.6.3 Preservation of data

The data in clinical trials should be kept according to GCP requirements.

9.7 Quality Control of Biological Specimens

9.7.1 Quality control of biological specimen collection and processing

Blood samples used for serum antibody detection should be centrifugally packed within 8 hours after collection. The hemolysis rate of serum is ≤2% and the error rate is ≤1%.

The sampling personnel should verify the basic information and procedures in the vaccination and method records, and disinfect the blood before taking blood. After specimen collection, the subject ID and specimen number must be indicated in relevant documents and blood collection tubes, and checked on the spot. After the samples are collected and numbered, the sampler should sign on the corresponding position of the original record book. Special circumstances of sample collection should be recorded accurately.

There is a special person who is responsible for the quality inspection of blood collection process, specimen quality and document filling. In case of wrong number, duplicate number or unqualified specimen, the person in charge of the site should be contacted immediately to remedy it in time.

The collected specimens should be properly kept, handed over to the laboratory blood-dividing personnel in time, and the handover records should be made. Medical wastes should be classified and placed as required, and handed over to the relevant person in charge for treatment in time.

9.7.2 Quality control of biological specimen transportation process

On-site specimens should be transported to specimen testing laboratory, and all specimens should be recorded according to regulations.

On-site logistics administrators should sort out the specimen transportation form before sending specimens: the contents should include specimen quantity, specimen box number, specimen number, etc. The paper version of the specimen transport list is transported with the specimen.

After receiving the specimen, the laboratory recipient should count the quantity and condition of the specimen, check whether the specimen is consistent with the waybill, check whether the specimen number is unique, and sign the specimen transportation form.

Temperature monitoring records shall be kept during the transportation of serum and saliva samples.

9.7.3 Quality control of biological specimen preservation

The temperature of all refrigerators related to the project should be monitored once in the morning and once in the evening. If the temperature is abnormal, the person in charge of the refrigerator needs to fill in the abnormal reasons and treatment measures on the temperature monitoring table. Including cold chain interruption alarm.

10 risk management plan

0.1 safety specification

Including important identified risks, important potential risks and important missing information. Make comprehensive consideration according to ADR collected in previous clinical studies, risks of medical treatment/intervention, class reactions, epidemiology of indications and target population, safety risks observed in non-clinical trials (including toxicology, drug interactions, etc.), people not studied in clinical trials, etc.

0.2 pharmacovigilance program

Refer to the research data, literature or reports on the safety of similar vaccines at home and abroad, closely monitor and report SUSAR and potential safety risks of research vaccines; If there are major safety risk warnings or reports, risk control plans should be made and necessary measures taken to protect the safety of the subjects. The safety monitoring data in clinical trials should be collected and analyzed regularly according to relevant requirements. In the analysis of monitoring data, the safety risk signals of drugs should be paid attention to. Based on the

analysis of the monitoring data, further identify the differences between the monitoring data and the safety information of drug instructions, analyze the occurrence of new and serious adverse reactions, discuss whether risk management measures should be taken, and put forward opinions on benefit risk assessment.

0.3 risk minimization measures

The main measures include: according to the information collected by long-term observation and follow-up, updating and revising the admission standard of the research scheme, notes in the researcher's manual, informed consent form, etc. in time; Additional risk minimization measures include grading the identified risks and issuing treatment suggestions, strengthening communication with the subjects, and giving the participating researchers corresponding training to pass on the treatment suggestions related to risk grading. In the trial scheme, a unified safety evaluation standard and method were formulated according to the corresponding guiding principles issued by the State Administration, and the safety of vaccines was actively monitored and followed up.

1 timetable

Total study time: This clinical trial is planned for about 8 months.

2 ethical approval

12.1 review and approval

The main researcher submits the clinical trial plan and all necessary additional documents to the ethics committee for initial review, and after the approval of the ethics committee, a written approval certificate is issued to the researcher.

At the same time, the researcher needs to provide the sample of informed consent form to the ethics committee, which will be approved by the ethics committee.

Before signing the informed consent form, volunteers have enough time to consider whether to participate in this experiment. Subjects have the opportunity to ask about the details of the experiment and get detailed answers. During the experiment, the subjects have the right to decide whether to withdraw from the experiment.

12.2 Supervise the following implementation processes

12.2.1 Informed consent

The method of selecting the subjects and whether the relevant information provided to the subjects is complete and understandable; Whether the method of obtaining informed consent is appropriate. During the whole experiment, the ethics committee should supervise whether there are ethical problems that harm the subjects, and whether the subjects get treatment or compensation when they are harmed by the experiment, and evaluate the degree of risks the subjects bear.

12.2.2 Confidentiality

Ensure the personal confidentiality of the subjects under the conditions of experiment,

biological sample collection, report and publication, etc. Samples only record ID number, random number and specimen number.

12.2.3 Potential hazards and minimization of hazards

1) Vaccination

Preliminary clinical trial data show that this vaccine is safe, and most adverse reactions are mild and transient. If the adverse reaction is determined to be related to vaccination (abscess at vaccination site), it will be treated in time according to relevant regulations. In case of life-threatening serious adverse events, immediately escort to the cooperative hospital for treatment through the green channel.

2) Specimen collection

Under strict supervision, experienced medical staff are trained to collect specimens according to prescribed procedures, so as to minimize the pain or danger suffered by the subjects.

In the whole process of the experiment, the ethics committee should supervise whether there are ethical problems that harm the subjects, whether the subjects get treatment or compensation when they are harmed by the experiment, and the corresponding measures, and evaluate the degree of risks the subjects bear.

13. Publication and publication of data

After the end of this clinical trial, if the test results need to be made public and/or published, the positive results and negative results will be made public and/or published together.

14. References

[1] Zhu, Feng-Cai et al. Immunogenicity and safety of a recombinant adenovirus type-5-vectored COVID-19 vaccine in healthy adults aged 18 years or older: a randomised, double-blind, placebo-controlled, phase 2 trial. The Lancet [J] 2020; S0140-6736(20)31605-6.

[2] Wang J, Deng C, Liu M, et al. Four doses of the inactivated SARS-CoV-2 vaccine redistribute humoral immune responses away from the Receptor Binding Domain. medRxiv; 2022. DOI: 10.1101/2022.02.19.22271215.

[3] Lu RJ, Zhao X, Li J, et al. Genomic characterisation and epidemiology of 2019 novel coronavirus: implications for virus origins and receptor binding. Lancet, 2020 https://doi.org/10.1016/S0140-6736(20)30251-8.

[4] Xie X, et al. Neutralization of N501Y mutant SARS-CoV-2 by BNT162b2 vaccine-elicited seraexternal icon. bioRxiv Posted January 7, 2021.

[5] Lauer SA, et al. The Incubation Period of Coronavirus Disease 2019 (COVID-19) From Publicly Reported Confirmed Cases: Estimation and Application [J]. Ann Intern Med, 2020, 172(9): 577-582.

[6] World Health Organzation. Modes of transmission of virus causing COVID-19: implications for IPC precaution Recommendations [EB/OL]. [2020-12-18].
